# Prenatal exposure to HIV pre-exposure prophylaxis and birth, growth, and social–emotional developmental outcomes throughout early childhood in Kenya: a prospective cohort study

**DOI:** 10.1016/S2214-109X(24)00471-6

**Published:** 2025-03

**Authors:** Laurén Gómez, John Kinuthia, Felix Abuna, Jared M Baeten, Julia Dettinger, Anna Larsen, Mary Marwa, Nancy Ngumbau, Ben Odhiambo, Pascal Omondi, Joshua Stern, Barbra A Richardson, Salphine Watoyi, Grace John-Stewart, Jillian Pintye

**Affiliations:** Department of Global Health, University of Washington, Seattle, WA, USA; Department of Global Health, University of Washington, Seattle, WA, USA; Department of Biobehavioral Nursing and Health Informatics, University of Washington, Seattle, WA, USA; Kenyatta National Hospital, Nairobi, Kenya; Department of Biobehavioral Nursing and Health Informatics, University of Washington, Seattle, WA, USA; Kenyatta National Hospital, Nairobi, Kenya; Department of Global Health, University of Washington, Seattle, WA, USA; Department of Global Health, University of Washington, Seattle, WA, USA; Department of Global Health, University of Washington, Seattle, WA, USA; Department of Biobehavioral Nursing and Health Informatics, University of Washington, Seattle, WA, USA; Kenyatta National Hospital, Nairobi, Kenya; Department of Biobehavioral Nursing and Health Informatics, University of Washington, Seattle, WA, USA; Kenyatta National Hospital, Nairobi, Kenya; Department of Biobehavioral Nursing and Health Informatics, University of Washington, Seattle, WA, USA; Kenyatta National Hospital, Nairobi, Kenya; Department of Biobehavioral Nursing and Health Informatics, University of Washington, Seattle, WA, USA; Kenyatta National Hospital, Nairobi, Kenya; Department of Global Health, University of Washington, Seattle, WA, USA; Department of Biostatistics, University of Washington, Seattle, WA, USA; Department of Biobehavioral Nursing and Health Informatics, University of Washington, Seattle, WA, USA; Kenyatta National Hospital, Nairobi, Kenya; Department of Global Health, University of Washington, Seattle, WA, USA; Department of Medicine, University of Washington, Seattle, WA, USA; Department of Pediatrics, University of Washington, Seattle, WA, USA; Department of Global Health, University of Washington, Seattle, WA, USA

## Abstract

**Background:**

As pre-exposure prophylaxis (PrEP) implementation continues to scale up among pregnant people, accruing safety data following prenatal PrEP exposure remains important. In this study, we aimed to evaluate the relationship between prenatal PrEP exposure and birth and infant or child outcomes.

**Methods:**

This prospective cohort study analysed data from the PrEP Implementation for Mothers in Antenatal Care study (NCT03070600). Participants were eligible for inclusion if they were currently pregnant, not currently using PrEP, were aged 15 years or older, planned to remain in the study area, were not enrolled in other studies, and did not have HIV or tuberculosis. Participants enrolled during pregnancy at 20 maternal and child health clinics in western Kenya and were followed up until 9 months postpartum. Those who reported taking PrEP at any antenatal visits were identified as prenatally PrEP exposed. In an extension cohort, participants and their children were followed up until 36 months postpartum. Infant anthropometry and social–emotional development using the Ages and Stages Questionnaire (ASQ-SE), second edition were assessed by trained study nurses. Among a subset of participants, we confirmed prenatal PrEP exposure using tenofovir diphosphate concentrations in dried blood spots. Perinatal outcomes (birth, growth, and neurodevelopment) were the primary outcomes assessed.

**Findings:**

Between Jan 15, 2018, and Jul 31, 2019, 4063 female individuals were enrolled and included in the analysis, of whom 558 (13·7%) used PrEP during pregnancy, initiating at a median of 26 weeks’ gestation (IQR 22–31) for a median duration of 9·6 weeks in pregnancy (5·7–15·0). Compared with PrEP-unexposed pregnancies, there was no difference in pregnancy loss (ie, miscarriage), stillbirth, preterm birth, or neonatal death among PrEP exposed pregnancies (all p>0·05). There were no differences in infant length or weight at 6 weeks, 6 months, and 9 months (all p>0·05) between children with and without prenatal PrEP exposure, including underweight, stunting, and wasting. Results were similar when analysed separately by trimester of PrEP initiation and duration on PrEP, and in a subset at 24 months, 30 months, and 36 months. Prenatal PrEP exposure was not associated with ASQ-SE scores at 24-months (p=0·12), 30-months (p=0·75), or 36-months (p=0·81). No differences in adverse perinatal and infant outcomes were found among Kenyan individuals with quantifiable prenatal tenofovir diphosphate exposure.

**Interpretation:**

We found no significant differences in adverse birth or infant or child outcomes for 3 years of follow-up by prenatal PrEP exposure status. These data support findings from previous studies that demonstrate the safety of oral PrEP use during pregnancy.

**Funding:**

The National Institutes of Health, National Institute of Allergy and Infectious Disease; Eunice Kennedy Shriver National Institute of Child Health and Human Development; the National Institute of Nursing Research; the University of Washington’s Center for AIDS Research Behavioral Sciences Core and Biometrics Core; and the Global Center for the Integrated Health of Women, Adolescents, and Children.

## Introduction

Globally, incident HIV infections continue to disproportionately occur among cisgender women, and there is evidence that HIV acquisition risk doubles during pregnancy and the postpartum period compared with that in other periods.^1,2^ Acute maternal HIV infection is associated with increased vertical transmission risk,^3^ making prevention of HIV among pregnant and breastfeeding people a global health priority.^4^ Based on a large body of safety data in women living with HIV who used tenofovir disoproxil fumarate for HIV treatment during pregnancy and breastfeeding,^5^ WHO recommends offering daily oral tenofovir disoproxil fumarate-based pre-exposure prophylaxis (PrEP) to pregnant people at substantial risk for HIV.^6^ Existing safety data of prenatal PrEP use among pregnant women are reassuring, finding no difference in pregnancy loss or preterm birth associated with PrEP use in pregnancy.^7^ However, few studies to date have follow-up beyond the perinatal period, and they rely on maternal self-report of PrEP adherence, which might not accurately measure infant PrEP exposure.^7^ Evaluating safety (including neurodevelopmental) outcomes beyond infancy using objective confirmation of maternal PrEP exposure could help to complete the safety profile for PrEP use during pregnancy. Findings regarding the effect antiretroviral exposure might have on child growth and development remain inconclusive; therefore, monitoring children exposed to antiretrovirals in their early childhood is warranted.^8^

In this study, we prospectively analysed data from cisgender women and their infants enrolled in the PrEP Implementation for Mothers in Antenatal Care (PrIMA) study to evaluate the relationship between prenatal PrEP exposure and birth and infant outcomes until 9 months of life.^9^ In an extension cohort, we evaluated growth and social–emotional development at 24, 30, and 36 months of life. We also evaluated perinatal and growth outcomes following maternal PrEP use in a subset of women, using confirmed tenofovir diphosphate concentrations in dried blood spots. Our overall objective was to expand evidence on the safety of PrEP use during pregnancy in settings with high HIV prevalence.

## Methods

### Study design

In this prospective cohort study, we used data from the PrIMA study, a cluster randomised trial of PrEP counselling strategies conducted between Jan 15, 2018, and Jan 15, 2021, in 20 mother and child health clinics in Homa Bay and Siaya counties, Kenya (NCT03070600). The study protocol has been described in detail previously.^9^

We also used data collected between Oct 26, 2020, and March 7, 2023, from an ongoing PrIMA Extension Study (PrIMA-X). The extension cohort was established to evaluate safety outcomes among mother–child pairs enrolled at four PrIMA sites to be followed up until the child’s fifth birthday with study visits every 6 months. Extension cohort participants were included in the analyses of outcomes beyond 24 months if they had data available on growth and neurodevelopmental outcomes.

The PrIMA and PrIMA-X study protocols were approved by the Kenyatta National Hospital, University of Nairobi Ethics and Research Committee and University of Washington Human Subjects Division. All participants provided written informed consent.

### Participants

Individuals attending antenatal care were eligible for enrolment if they were: currently pregnant, not currently using PrEP, aged 15 years or older, planned to remain in the area for at least 9 months postpartum and receive postnatal and infant care at the study facility, were not currently enrolled in other studies, and if they did not have HIV or tuberculosis. Following enrolment, pregnant people were counselled on PrEP as part of routine antenatal care, either universally or after undergoing HIV risk screening, and identified as at risk of HIV infection. People enrolled at any gestational age during pregnancy and were followed up every month until the end of their pregnancy and at 6 weeks, 14 weeks, 6 months, and 9 months postpartum, regardless of their PrEP use.

Participant sex was collected via self-report, with the options of male or female.

Participants were included in the primary analysis if they had complete information on their PrEP use and birth outcomes. Those who initiated PrEP postpartum, HIV seroconverted, had multiple pregnancies, or were missing information on PrEP use in pregnancy or birth outcomes were excluded.

### Procedures

At enrolment, demographic, clinical, and psychosocial characteristics were ascertained by trained study staff using tablet-based data capture systems via the REDCap mobile application.^10^ Sexual history, behaviours associated with HIV acquisition, and partner HIV status were collected at enrolment and subsequent visits. Oral tenofovir disoproxil fumarate-based PrEP was offered at enrolment and was available at every follow-up visit. People who initiated PrEP during the study received PrEP counselling and PrEP refill at all subsequent visits. At follow-up visits, PrEP use and adherence was ascertained via self-report. Pregnancy end date and gestational age at pregnancy end were ascertained by record abstraction, where available, or via direct interview, either at the first postnatal care visit or via telephone call conducted by study nurses on the expected date of delivery, and weekly thereafter until pregnancy end was confirmed. Birthweight, birth length, and information on congenital anomalies were collected at the first postnatal visit. Infant weight and length were measured by study nurses trained in anthropometry at every postnatal visit. In the extension cohort, study nurses took anthropometric measurements of children at every study visit and assessed social–emotional development using the Ages and Stages Social-Emotional Questionnaire (ASQ-SE, second edition), an early developmental screener.^11^

Dried blood spots were collected for tenofovir diphosphate quantification by study nurses who received standardised training on dried blood spot collection via fingerstick. Dried blood spots were transported to a central −20°C freezer for storage within 48 h after collection. A subset of participants who self-reported PrEP use in the previous 30 days at antenatal visits were randomly selected and tenofovir diphosphate concentrations were measured in red blood cells from dried blood spots using validated ultra-performance liquid chromatography-tandem mass spectrometry at the University of Colorado Aurora (CO, USA).^12^ Values below the lower limit of quantification for tenofovir diphosphate (25 fmol per sample) were considered unquantifiable.^12^ Syphilis status was assessed by rapid plasma reagin test when available; otherwise, a dual HIV and syphilis test was used.

In the primary analysis, PrEP exposure was defined as any self-reported PrEP pill taking during pregnancy. For sub-analyses, the trimester of PrEP exposure was assessed based on gestational age at PrEP initiation, using fundal height and last menstrual period; duration of PrEP use during pregnancy was defined as weeks since PrEP initiation. Among the randomly selected subset, confirmed PrEP exposure during pregnancy was defined as having quantifiable tenofovir diphosphate in dried blood spots for exploratory analyses. Those who were randomly selected but did not have detectable tenofovir diphosphate were excluded, without replacement.

### Outcomes

Perinatal outcomes, the analysis’ primary outcome, included gestational age at birth, preterm birth (<37 weeks’ gestation), any congenital malformation (ie, cleft lip, club foot, jointed fingers or toes, and extra fingers or toes), pregnancy loss (ie, miscarriage; <20 weeks’ gestation), stillbirth (≥20 weeks’ gestation), and neonatal death (<28 days after birth). Study staff received standardised multi-day training on conducting anthropometric and neurodevelopmental measurements, which included theoretical and didactic sessions and observed practice. WHO weight-for-age, length-for-age, weight-for-length, and small-for-gestational-age Z scores were calculated; underweight, stunting, wasting, and small for gestational age were defined as Z scores less than −2 SD below the mean.^13^ Low birthweight was defined as less than 2·5 kg among full term infants, and small for gestational age as less than the 10th percentile for birthweight for their gestational age at birth. ASQ-SE scores were calculated and cutoffs indicating referral for further assessment were defined as greater than 85 at 30 months and greater than 105 at 36 months, following the manual. People who experienced miscarriage, stillbirth, or death of a child remained enrolled, allowing for consistent ascertainment of those outcomes.

### Statistical analysis

We used generalised estimating equations models clustered by site to evaluate the differences in baseline characteristics by PrEP exposure status, and the association of birth, infant, and early childhood outcomes by exposure to antenatal PrEP (yes or no), trimester of PrEP initiation (<14 weeks, 14–27 weeks, or ≥27 weeks), and duration on PrEP (weeks since PrEP initiation date <4 weeks, 4–12 weeks, and >12 weeks). Gaussian linear regression models with identity-link were used for continuous outcomes and Poisson regression with a log-link was used for binary outcomes. Independent correlation structure and robust SEs were used for all models. Outcomes analysed at 24–36 months were not clustered by site due to small cluster size. Multivariate models were adjusted for maternal age, primigravida, gestational age at enrolment, partner HIV status, and maternal syphilis a priori based on their association with adverse perinatal, infant outcomes, or PrEP uptake in the literature in different combinations.^14–16^ PrEP uptake and perinatal and infant outcomes were not associated with randomisation group in the parent study and therefore we did not adjust for randomisation group.^17^ In exploratory analyses, the frequency of adverse perinatal outcomes was compared between individuals with and without quantifiable tenofovir diphosphate concentrations. We conducted a sensitivity analysis using adherence benchmarks established in IMPAACT 2009 for pregnant people who used daily oral tenofovir disoproxil fumarate or emtricitabine-based PrEP for more than 4 weeks.^18^ We also evaluated the association of prenatal PrEP exposure and ASQ-SE scores. All analyses were conducted in Stata 17.

### Role of the funding source

The funders of the study had no role in study design, data collection, data analysis, data interpretation, or writing of the report.

## Results

Between Jan 15, 2018, and July 31, 2019, 4063 female individuals met inclusion criteria and were included in the analysis (91% of total PrIMA participants, appendix 2 p 1). At enrolment, the median maternal age was 24 years (IQR 21–28) and median gestational age was 24 weeks (20–30, table 1); 1050 (25·8%) were primigravida. 3392 (83·5%) participants were married. 1270 (31·3%) reported not knowing their partner’s HIV status and 159 (3·9%) had partners who were known to be living with HIV. The median years of education was 10 (IQR 8–12) and 601 (14·8%) had some form of employment. 305 (7·5%) reported experiencing intimate partner violence (IPV) in the 6 months before enrolment, 399 (9·8%) experienced household crowding, defined as more than 3 people per room in their residence, and 526 (12·9%) had previous history of pregnancy loss; 42 (1·0%) reported a previous premature birth.

Out of a total of 4063 pregnancies included in the analysis, 3971 (97·7%) resulted in livebirths. Of these neonates, 1727 (43·5%) were male and 1800 (45·3%; some data missing) were female.

In total, 558 (13·7%) participants self-reported PrEP use at some point during pregnancy with a median gestational age at PrEP initiation of 26 weeks (IQR 22–31). Median duration of PrEP use during pregnancy was 9·6 weeks (5·7–15·0). Compared with individuals who did not use PrEP during pregnancy, those who used PrEP were more likely to report having a partner who was known to be living with HIV (108 [19·4% *vs* 51 [1·5%] of 3505, p<0·0001) or a partner of unknown HIV status (227 [40·7%] *vs* 1043 [29·8%], p<0·0001; table 1). Participants who used PrEP in pregnancy also more frequently had characteristics associated with HIV acquisition, including engaging in transactional sex (p=0·0020), having recent sexually transmitted infection diagnosis (p<0·0001), including syphilis (p=0·0009), and experiencing IPV in the past 6 months (p<0·0001).

875 (21·5%) adverse perinatal outcomes occurred, with 12 (1·3%) miscarriages, 31 (3·5%) stillbirths, 755 (86·3%) preterm births, 56 (6·4%) reports of small birthweight, and 21 (2·4%) reports of congenital malformation, which were not mutually exclusive. There were no significant differences in the frequency of miscarriage (adjusted prevalence ratio [aPR] 1·81, 95% CI 0·26 to 12·35, p=0·55), stillbirth (0·77, 0·29 to 2·07, p=0·61), and preterm birth (0·95, 0·74 to 1·21, p=0·67) between participants who did and did not use PrEP in pregnancy (table 2). PrEP exposed and unexposed infants had similar birthweight (exposed 3·3 kg *vs* unexposed 3·4 kg, adjusted beta coefficient [aβ] −0·04, 95% CI −0·10 to 0·03, p=0·28) and birth length (50 cm for both, −0·03, −0·69 to 0·77, p=0·95). The proportion of infants who were born small for their gestational age was similar among the two groups (aPR 1·10, 95% CI 0·77 to 1·56, p=0·60). Few congenital malformations were reported and the incidence did not statistically differ between infants who were and were not perinatally exposed to PrEP (2·23, 0·94 to 5·28, p=0·068). There was also no significant difference in the frequency of neonatal deaths between PrEP exposed and unexposed infants (1·26, 0·59 to 2·72, p=0·55).

Retention was high, with 3908 (96·2%) of 4063 individuals completing the study at 9 months postpartum. Overall, 2919 (71·8%) infants had complete growth measurements at 6 weeks, 2247 (55·3%) at 6 months, and 2213 (54·5%) at 9 months and were included in the infant growth analysis separately for each timepoint. Median infant weight was similar at 6 weeks (both 5·0 kg, aβ=0·01, 95% CI −0·09 to 0·10, p=0·90) and 9 months (both 8·6 kg, 0·05, −00·06 to 0·16, p=0·34) between PrEP exposed and unexposed groups. At 6 months, PrEP exposed infants had slightly higher weight (7·8 kg *vs* 7·7 kg, aβ=0·18, 0·06 to 0·29, p=0·0039). There were no differences in median infant length at 6 weeks (exposure 55·1 cm *vs* no exposure 55·0 cm, aβ −0·49, −01·76 to 0·78, p=0·43), 6 months (both 66·0 cm, 0·29, aβ −0·44 to 1·01, p=0·42), and 9 months (70·5 *vs* 70·0 cm, aβ −00·41, −01·77 to 0·95, p=0·54). Weight-for-age and length-for-age Z scores did not significantly differ by PrEP exposure at any timepoint. Prenatal PrEP exposure was not associated with underweight, stunting, or wasting at any age (table 3). Results were similar when analysed separately by trimester of PrEP initiation and weeks of duration on PrEP in pregnancy (appendix 2 pp 2–9).

Overall, 669 mother–child pairs from the PrIMA Extension cohort met inclusion criteria and were included in the extended analysis. Median child age was 26 months (IQR 22 to 32) at enrolment into the extension cohort; 116 (17·3%) had any PrEP exposure during pregnancy, initiating PrEP at a median of 27 weeks’ gestation (IQR 21 to 31) and during pregnancy used PrEP for a median duration of 3 months (1 to 4). Many participants continued PrEP postnatally, with 63 (54·3%) of the 116 individuals who initiated PrEP during pregnancy continuing to use PrEP for 9 months postpartum. At the 24-month visits, there was no difference in mean weight (mean difference −0·04 kg, 95% CI −0·47 to 0·39, p=0·86), mean height (−0·57 cm, −1·74 to 0·60, p=0·34), frequency of underweight (aPR 0·44, 95% CI 0·05 to 3·83, p=0·46), frequency of stunting (0·53, 0·16 to 1·77, p=0·31), or frequency of wasting (0·75, 0·21 to 2·60, p=0·65) between children with and without prenatal PrEP exposure. Results were similar at the 30-month and 36-month visits (table 4), except for the 30-month frequency of wasting, which was higher among PrEP exposed children than non-exposed children (2·33, 1·20 to 4·52, p=0·012). Prenatal PrEP exposure was not associated with overall ASQ-SE scores at 24 months (27·73, −7·87 to 62·33, p=0·12), 30 months (−1·50, −18·60 to 13·47, p=0·75), or 36 months (1·10, −8·02 to 10·23; p=0·81). Similarly, after dichotomising ASQ-SE scores by standard cutoffs, there was no association between prenatal PrEP exposure and adverse social–emotional development at 24 months (aPR=1·55, 95% CI 0·38 to 6·26, p=0·54), 30 months (0·85, 0·33 to 2·24, p=0·75) or 36 months (0·82, 0·40 to 1·69, p=0·60; table 5).

A subset of 103 (18·5%) of the 558 participants who initiated PrEP were randomly selected for dried blood spot testing and had detectable tenofovir diphosphate during pregnancy; these participants were compared with 3505 participants who did not use PrEP during pregnancy. Compared with PrEP-unexposed individuals, those with confirmed tenofovir diphosphate exposure during pregnancy had similar frequencies of stillbirth (aPR 1·03, 95% CI 0·12–8·93, p=0·98), preterm birth (0·92, 0·58–1·47, p=0·73), small for gestational age (1·41, 0·78–2·54, p=0·26), and neonatal death (0·69, 0·09–5·26, p=0·72; appendix 2 pp 9–10). At 9 months, there was no association between prenatal PrEP exposure and frequency of underweight (0·77, 0·23–2·61, p=0·68), stunting (0·42, 0·06–3·06, p=0·39), or wasting (1·16, 0·39–3·52, p=0·79); results were similar at 6 weeks and 6 months (appendix 2 pp 11–12). Results were consistent comparing the 72 (69·9%) participants with tenofovir diphosphate concentrations indicative of two or more doses per week during pregnancy compared with those who did not use PrEP during pregnancy (appendix 2 pp 13–15).

## Discussion

In this large prospective safety evaluation of prenatal oral PrEP use, maternal PrEP use during pregnancy, regardless of trimester of PrEP initiation or duration of prenatal use, was not associated with differences in birth or infant outcomes for the first 9 months of life among Kenyan mother–infant pairs. Similar to previous safety data that relied on self-reported PrEP use, we found no differences in adverse perinatal outcomes among PrEP exposed pregnancies, compared with pregnancies without PrEP use, confirmed with a biological measure. Retention in the study was high and in a subset of participants enrolled in an extension cohort, there were also no differences in growth or social–emotional development at 24, 30, and 36 months among children who did and did not have prenatal PrEP exposure. Our results support previous data indicating safety of prenatal PrEP use and provide evidence for future scale-up of PrEP delivery in this population. To our knowledge, this is the largest study of PrEP use during pregnancy to date, with the longest follow-up, and it is one of the only safety evaluations to include confirmation of maternal PrEP use using quantified tenofovir diphosphate exposure.

Programmatic PrEP delivery to pregnant individuals is ongoing in Kenya;^19^ however, uncertainty about safety of prenatal PrEP use has hindered PrEP implementation among pregnant populations in some settings with high HIV burden. For example, a paucity of comprehensive safety data delayed PrEP roll-out in South Africa.^20^ Currently, most studies contributing data to safety considerations for prenatal PrEP use are from women with HIV using tenofovir disoproxil fumarate as part of combination antiretroviral therapy (ART) regimens for HIV or hepatitis B treatment.^21^ Insufficient data are available on the outcomes of PrEP use throughout pregnancy and the postpartum period, and few studies assess longitudinal outcomes among perinatally exposed infants.^7^ In initial PrEP efficacy trials, pregnancy was an exclusion criterion and people who became pregnant during the trials discontinued PrEP at pregnancy detection. Therefore, these studies only provide data on short-term, early first-trimester exposure, which might not be representative of all pregnant individuals in these settings. Additionally, existing studies among infants prenatally exposed to PrEP have less than 1 year of follow-up and have not assessed other longer-term developmental outcomes, including neurodevelopment. Previous studies have had smaller sample sizes and often relied on abstracting infant outcomes from medical records, which in some cases can be incomplete or inaccurate. Expanding on previous studies and evaluating outcomes beyond infancy following maternal PrEP helps to complete the safety profile for PrEP use during pregnancy.

Previous studies on tenofovir disoproxil fumarate and infant safety outcomes have predominantly come from studies among women with HIV, and thus involve antiretroviral and HIV exposure to infants, rather than purely antiretroviral exposure, which we have examined in this study. In the PROMISE trial, use of tenofovir disoproxil fumarate-based ART in mothers with HIV resulted in more frequent severe adverse pregnancy outcomes and higher rates of preterm delivery (before 34 weeks) than use of zidovudine-based ART.^22^ In the SMARTT cohort, again in women with HIV, tenofovir disoproxil fumarate use in pregnancy was associated with reduced bone mineral content in neonates compared with exposure to other antiretrovirals, although tenofovir concentrations in meconium were not associated with infant weight, length, or bone mineral content.^23^ A study using dual-energy x-ray absorptiometry suggests that any prenatal antiretroviral exposure among infants of women with HIV might lead to decrement in neonatal bone mineral density.^24^ Tenofovir disoproxil fumarate use during pregnancy has been associated with lower length-for-age Z scores at 1 year compared with those without tenofovir disoproxil fumarate use among children who are HIV-exposed but uninfected.^24^ Whether these changes are clinically relevant is unclear. Importantly, these data from women with HIV are complicated by concomitant ART drugs and HIV disease and might not necessarily reflect the safety of tenofovir disoproxil fumarate use among mothers who are HIV negative. Our findings suggest that prenatal PrEP use is not associated with adverse growth and development among mother–infant pairs who are HIV negative. WHO guidelines strongly advocate for active surveillance of mother and infant outcomes during PrEP use in pregnancy and breastfeeding as part of PrEP programmes and call for data on longer term infant outcomes following maternal PrEP use.^6^

Neurodevelopmental deficits have been seen in children who are HIV-exposed and uninfected, compared with children without HIV exposure; however, the role of antiretroviral drug exposure in these deficits is unclear. Evidence regarding regimen-specific effects are inconsistent and sparse. Although many studies support the safety of tenofovir disoproxil fumarate use during pregnancy,^7^ prenatal tenofovir disoproxil fumarate exposure among children who are HIV-exposed and uninfected has been associated with lower performance on social–emotional domains.^25^ The current study found no differences in social–emotional development between children with and without prenatal exposure to PrEP.

To our knowledge, this study is one of the first to assess the safety of peripartum PrEP exposure using adherence biomarkers.^26^ Existing safety studies of prenatal PrEP use have primarily relied on maternal self-report of oral PrEP adherence, which might not accurately measure infant PrEP exposure.^7^ Self-reported adherence has been shown to correlate poorly with objectively measured adherence in PrEP trials.^27^ Biological measures, in contrast, can objectively assess adherence, but are less commonly implemented due to cost and logistical constraints.^28^ In a subset of participants with quantified tenofovir exposure from collected biological specimens, we found no differences in adverse perinatal outcomes among Kenyan individuals with prenatal PrEP exposure.

A limitation of this study is that we relied on last menstrual period and fundal height for determining gestational age. This approach to measuring gestational age is standard of care in this setting and previous studies have shown moderate correlation between these measures and ultrasound.^29^ The study observed low rates of pregnancy loss, which might indicate survivorship bias since individuals in the parent study enrolled at a median of 24 weeks’ gestation, thus earlier pregnancy losses were not captured. However, rates were consistent with local estimates reported in 2022.^30^ This gestational age aligns with typical timing of presentation to antenatal care in Kenya.^31^ Results might not be generalisable to those who initiate PrEP before conception or those who are not retained in care. Rare outcomes, including congenital anomalies, will require future larger surveillance evaluations. Although the prevalence of preterm birth was higher than global estimates, our results align with 2022 estimates from the region.^32^ Cumulative pregnancy loss prevalence in our study was similar to current regional and Kenyan estimates.^33^

Understanding the effects of perinatal PrEP use on infant outcomes is crucial for informing future scale-up of PrEP delivery. Additional long-term follow-up including school ages will be useful to elucidate the effect of perinatal PrEP exposure on child development. A more comprehensive safety profile including broader neurodevelopmental assessment and bone density measurements remains important. Data from individuals who initiate PrEP before conception in programatic settings are needed to confirm safety of PrEP use during pregnancy. However, existing data on periconception PrEP use is reassuring.^34^

In summary, among Kenyan mother–child pairs followed up from pregnancy until early childhood, we found no differences in adverse perinatal, growth, or social–emotional development between children with versus without prenatal PrEP exposure. Our findings suggest that oral PrEP use during pregnancy does not influence birth and infant outcomes before 36 months postpartum. Our data support findings from previous studies that show safety of PrEP use during pregnancy and, importantly, this study contributes to the sparse data available on early childhood outcomes following antiretroviral exposure without the confounder of HIV exposure.

## Supplementary Material

1

2

3

## Figures and Tables

**Table 1:** Characteristics of participants included in analysis by PrEP exposure

	Never PrEP exposed (n=3505)	Any PrEP exposure during pregnancy (n=558)

Age (years)*	23 8 (20·8–28·0)	25·0 (21·0–30·0)
Age category*		
<24 years	2065 (58·9%)	276 (49·5%)
24·35 years	1283 (36·6%)	240 (43·0%)
≥35 years	155 (4·4%)	42 (7·5%)
Missing	2 (0·1%)	0
Currently married	2911 (83·1%)	481 (86·2%)
Missing	40 (1·1%)	7 (1·3%)
Currently polygamous*†	313 (10·8%)	113 (23·6%)
Missing	17 (0·5%)	3 (0·5%)
Gestational age at enrolment (weeks)	24·0 (20·0–30·0)	24·0 (18·6–28·0)
Missing	0	0
Attended four or more antenatal care visits	2459 (70·2%)	411 (76·4%)
Missing	133 (3·8%)	20 (3·6%)
Positive for syphilis on rapid plasma reagin or HIV-syphilis dual test*	29 (0·8%)	13 (2·3%)
Missing	58 (1·7%)	17 (3·1%)
Primigravida*	961 (27·4%)	89 (15·9%)
Missing	15 (0·4%)	3 (0·5%)
Number of living children*	1·0 (0·0–2·0)	2·0 (1·0–3·0)
Missing	78 (2·2%)	9 (1·6%)
Previous history of pregnancy loss (ie, miscarriage) and stillbirth	438 (12·5%)	88 (15·8%)
Missing	17 (0·5%)	11 (2·0%)
Previous premature birth (<37 weeks)	30 (0·9%)	12 (2·2%)
Missing	0	0
Infant sex		
Female	1561 (44·5%)	240 (43·0%)
Male	1480 (42·2%)	251 (45·0%)
Missing	464 (13·2%)	67 (12·0%)
Breastfeeding at 6 months‡	2560 (95·4%)	371 (94·6%)
Missing	158 (4·5%)	52 (9·3%)
Introduced any other food or drink besides breastmilk yet§	1606 (62·9%)	255 (68·7%)
Missing	7 (0·2%)	0
Number of lifetime sexual partners*	2·0 (2·0–3·0)	3·0 (2·0–4·0)
Missing	8 (0·2%)	4 (0·7%)
HIV status of current sexual partners*		
Negative	2360 (67·3%)	216 (38·7%)
Unknown	1043 (29·8%)	227 (40·7%)
Positive	51 (1·5%)	108 (19·4%)
No partner	45 (1·3%)	4 (0·7%)
Missing	6 (0·2%)	3 (0·5%)
Engaged in sex in exchange of money or favours in the last 6 months*	54 (1·5%)	19 (3·4%)
Missing	14 (0·4%)	4 (0·7%)
Diagnosed with or treated for an STI in the last 6 months*	72 (2·1%)	28 (5·0%)
Missing	12 (0·3%)	4 (0·7%)
Forced to have sex in the last 6 months*	172 (4·9%)	53 (9·5%)
Missing	11 (0·3%)	5 (0·9%)
Moderate to severe risk for intimate partner violence (HITS scale)*	222 (6·4%)	83 (14·9%)
Missing	18 (0·5%)	2 (0·4%)
High risk by NASCOP assessment*	384 (11·0%)	120 (21·5%)
Missing	0	0
High risk by empirical HIV risk score*	1143 (32·6%)	354 (63·4%)
Missing	0	0
High risk score by either assessment*	1334 (38·1%)	382 (68·5%)
Missing	0	0

Data are n (%) or median (IQR). HITS=Hurt, Insult, Threaten, Scream. NASCOP=National AIDS and STI Control Program. PrEP=pre-exposure prophylaxis. STI=sexually transmitted infection.

* p<0·01 determined by generalised estimating equation family-gaussian, link-identity, and correlation-independent for continuous variables and family-poisson, link-log, and correlation-independent for binary variables.

† Among those who were married (n=2911 for no exposure and n=481 for exposure).

‡ Among those who attended their 6-month visit (n=2842 for no exposure and n=444 for exposure).

§ Among those who attended their 6-month visit and were currently breastfeeding at 6 months (n=2560 for no exposure and n=371 for exposure).

**Table 2:** Perinatal outcomes by PrEP exposure status

	Never PrEP exposed	Any PrEP exposure during pregnancy	Unadjusted generalised estimating equations regression		Adjusted generalised estimating equations regression*	
			Coefficient† (95% CI)	p value	Coefficient† (95% CI)	p value*

Gestational age at pregnancy end (weeks)	38·0 (37·0 to 39·0)	38·0 (37·1 to 39·0)	−0·01 (−0·30 to 0·29)	0·97	−0·02 (−0·30 to 0·27)	0·91
Preterm birth (<37 weeks’ gestation)						
No	2783/3444 (80·8%)	461/555 (83·1%)	1 (Ref)	··	1 (Ref)	··
Yes	661/3444 (19·2%)	94/555 (16·9%)	0·88 (0·71 to 1·10)	0·26	0·95 (0·74 to 1·21)	0·67
Pregnancy loss (ie, miscarriage; <20 weeks’ gestation)						
No	831/841 (98·8%)	154/156 (98·7%)	1 (Ref)	··	1 (Ref)	··
Yes	10/841 (1·2%)	2/156 (1·3%)	1·08 (0·24 to 4·89)	0·92	1·81 (0·26 to 12·35)	0·55
Stillbirth (≥20 weeks’ gestation)						
No	815/841 (96·9%)	151/156 (96·8%)	1 (Ref)	··	1 (Ref)	··
Yes	26/841 (3·1%)	5/156 (3·2%)	1·04 (0·43 to 2·48)	0·94	0·77 (0·29 to 2·07)	0·61
Birthweight (kg)	3·4 (3·0 to 3·6)	3·3 (3·0 to 3·6)	−0·02 (−0·08 to 0·05)	0·55	−0·04 (−0·10 to 0·03)	0·28
Birthweight (<2·5 kg)						
No	2110/2160 (97·7%)	384/390 (98·5%)	1 (Ref)	··	1 (Ref)	··
Yes	50/2160 (2·3%)	6/390 (1·5%)	0·66 (0·36 to 1·22)	0·19	0·68 (0·39 to 1·18)	0·17
Small for gestational age						
No	1944/2156 (90·2%)	348/390 (89·2%)	1 (Ref)	··	1 (Ref)	··
Yes	212/2156 (9·8%)	42/390 (10·8%)	1·10 (0·78 to 1·53)	0·60	1·10 (0·77 to 1·56)	0·60
Congenital malformation‡						
No	3414/3430 (99·5%)	536/541 (99·1%)	1 (Ref)	··	1 (Ref)	··
Yes	16/3430 (0·5%)	5/541 (0·9%)	1·98 (0·88 to 4·44)	0·096	2·23 (0·94 to 5·28)	0·068
Neonatal death						
No	3375/3430 (98·4%)	531/541 (98·2%)	1 (Ref)	··	1 (Ref)	··
Yes	55/3430 (1·6%)	10/541 (1·9%)	1·15 (0·52 to 2·55)	0·73	1·26 (0·59 to 2·72)	0·55

Data are n (%) or median (IQR) unless otherwise specified. The overall n for never PrEP exposed was 3505 and the overall n for any PrEP exposure during pregnancy was 558. PrEP=pre-exposure prophylaxis.

* Adjusted for maternal age at enrolment, primigravida at enrolment, gestational age at enrolment, partner HIV status at enrolment, and syphilis status at enrolment.

† Prevalence ratios are shown for binary outcomes and β-coefficients are shown for continuous outcomes.

‡ Congenital anomalies included club foot, congenital hip dislocation, jointed fingers or toes, and extra fingers or toes.

**Table 3:** Infant growth outcomes at 6 weeks, 6 months, and 9 months postpartum by PrEP exposure

	Never PrEP exposed	Any PrEP exposure during pregnancy	Unadjusted generalised estimating equations regression		Adjusted generalised estimating equations regression*	
			Coefficient†(95% CI)	p value	Coefficient†(95% CI)	p value*

**6-week analysis**						
Participants attending 6-week assessment	2524/3505 (72·0%)	395/558 (70·8%)	··	··	··	··
Weight (kg)	5·0 (4·5 to 5·4)	5·0 (4·5 to 5·4)	0·01 (−0·07 to 0·09)	0·80	0·01 (−0·09 to 0·10)	0·90
Absolute WAZ	0·3 (−0·4 to 0·9)	0·3 (−0·4 to 0·8)	−0·02 (−0·14 to 0·09)	0·66	−0·03 (−0·16 to 0·09)	0·58
Underweight (less than −2 WAZ)						
No	2086/2151 (97·0%)	284/291 (97·6%)	1 (Ref)	··	1 (Ref)	··
Yes	65/2151 (3·0%)	7/291 (2·4%)	0·80 (0·44 to 1·45)	0·45	0·84 (0·45 to 1·59)	0·60
Length (cm)	55·0 (54·0 to 57·0)	55·1 (54·0 to 57·0)	−0·50 (−1·68 to 0·68)	0·39	−0·49 (−1·76 to 0·78)	0·43
Absolute LAZ	−0·2 (−1·1 to 0·7)	−0·2 (−0·9 to 0·7)	0·5 (−0·15 to 0·25)	0·62	0·04 (−0·16 to 0·24)	0·68
Stunting (less than −2 LAZ)						
No	1907/2108 (90·5%)	257/286 (89·9%)	1 (Ref)	··	1 (Ref)	··
Yes	201/2108 (9·5%)	29/286 (10·1%)	1·06 (0·71 to 1·59)	0·76	1·20 (0·80 to 1·80)	0·37
Absolute WLZ	0·6 (−0·3 to 1·7)	0·6 (−0·2 to 1·6)	−0·02 (−0·23 to 0·18)	0·83	−0·01 (−0·26 to 0·23)	0·92
Wasting (less than −2 WLZ)						
No	1949/2070 (94·2%)	266/280 (95·0%)	1 (Ref)	··	1 (Ref)	··
Yes	121/2070 (5·9%)	14/280 (5·0%)	0·86 (0·44 to 1·66)	0·64	0·82 (0·41 to 1·62)	0·56
**6-month analysis**						
Participants attending 6-month assessment	1945/3505 (55·5%)	302/558 (54·1%)	··	··	··	··
Weight (kg)	7·7 (7·0 to 8·5)	7·8 (7·2 to 8·7)	0·16 (0·04 to 0·29)	0·013	0·18 (0·06 to 0·29)	0·0039
Absolute WAZ	0·1 (−0·7 to 0·9)	0·2 (−0·4 to 1·1)	0·19 (0·04 to 0·34)	0·016	0·20 (0·05 to 0·35)	0·013
Underweight (less than −2 WAZ)						
No	1678/1737 (96·6%)	249/255 (97·7%)	1 (Ref)	··	1 (Ref)	··
Yes	59/1737 (3·4%)	6/255 (2·4%)	0·69 (0·31 to 1·57)	0·38	0·65 (0·28 to 1·50)	0·31
Length (cm)	66·0 (64·0 to 68·0)	66·0 (64·0 to 69·0)	0·35 (−0·37 to 1·07)	0·32	0·29 (−0·44 to 1·01)	0·42
Absolute LAZ	−0·3 (−1·1 to 0·6)	−0·2 (−1·0 to 0·8)	0·07 (−0·14 to 0·27)	0·50	0·04 (−0·19 to 0·26)	0·73
Stunting (less than −2 LAZ)						
No	1575/1725 (91·3%)	233/254 (91·7%)	1 (Ref)	··	1 (Ref)	··
Yes	150/1725 (8·7%)	21/254 (8·3%)	0·95 (0·66 to 1·37)	0·79	1·04 (0·75 to 1·45)	0·81
Absolute WLZ	0·4 (−0·5 to 1·4)	0·5 (−0·4 to 1·5)	0·11 (−0·08 to 0·30)	0·23	0·16 (−0·04 to 0·36)	0·12
Wasting (less than −2 WLZ)						
No	1645/1712 (96·1%)	241/248 (97·2%)	1 (Ref)	··	1 (Ref)	··
Yes	67/1712 (3·9%)	7/248 (2·8%)	0·72 (0·34 to 1·54)	0·40	0·66 (0·28 to 1·55)	0·34
**9-month analysis**						
Participants attending 9-month assessment	1908/3505 (54·4%)	305/558 (54·7%)	··	··	··	··
Weight (kg)	8·6 (7·9 to 9·6)	8·6 (8·0 to 9·5)	0·03 (−0·08 to 0·13)	0·60	0·05 (−0·06 to 0·16)	0·34
Absolute WAZ	0·1 (−0·7 to 1·0)	0·1 (−0·5 to 0·9)	0·04 (−0·06 to 0·14)	0·45	0·04 (−0·07 to 0·16)	0·44
Underweight (less than −2 WAZ)						
No	1671/1738 (96·1%)	268/276 (97·1%)	1 (Ref)	··	1 (Ref)	··
Yes	67/1738 (3·9%)	8/276 (2·9%)	0·75 (0·39 to 1·46)	0·40	0·75 (0·37 to 1·53)	0·44
Length (cm)	70·0 (68·0 to 72·0)	70·5 (68·7 to 72·0)	−0·28 (−1·64 to 1·07)	0·67	−0·41 (−1·77 to 0·95)	0·54
Absolute LAZ	−0·4 (−1·3 to 0·5)	−0·2 (−1·1 to 0·6)	0·10 (−0·14 to 0·34)	0·39	0·07 (−0·16 to 0·30)	0·56
Stunting (less than −2 LAZ)						
No	1544/1701 (90·8%)	249/267 (93·3%)	1 (Ref)	··	1 (Ref)	··
Yes	157/1707 (9·2%)	18/267 (6·7%)	0·73 (0·44 to 1·21)	0·22	0·81 (0·49 to 1·32)	0·40
Absolute WLZ	0·4 (−0·5 to 1·3)	0·4 (−0·5 to 1·3)	−0·02 (−0·18 to 0·14)	0·81	0·03 (−0·15 to 0·22)	0·71
Wasting (less than −2 WLZ)						
No	1631/1691 (96·5%)	259/267 (97·0%)	1 (Ref)	··	1 (Ref)	··
Yes	60/1691 (3·6%)	8/267 (3·0%)	0·84 (0·43 to 1·67)	0·63	0·79 (0·36 to 1·70)	0·54

Data are n (%) or median (IQR) unless otherwise specified. The overall N for never PrEP exposed was 3505 and the overall N for any PrEP exposure during pregnancy was 558. PrEP=pre-exposure prophylaxis. LAZ=length-for-age Z score. WAZ=weight-for-age Z score. WLZ=weight-for-length Z score.

* Adjusted for maternal age at enrolment, primigravida at enrolment, gestational age at enrolment, partner HIV status at enrolment, and syphilis status at enrolment.

† Prevalence ratios are shown for binary outcomes and β-coefficients are shown for continuous outcomes.

**Table 4:** Early childhood growth outcomes at 24, 30, and 36 months by prenatal PrEP exposure

	Never PrEP exposed	Any PrEP exposure during pregnancy	Unadjusted regression		Adjusted regression*	
			Coefficient† (95% CI)	p value	Coefficient† (95% CI)	p value*

**24-month analysis**						
Participants attending 24-month assessment	282/553 (51·0%)	61/116 (52·6%)	··	··	··	··
Weight (kg)	11·5 (10·5 to 12·6)	11·3 (10·3 to 13·0)	−0·03 (−0·44 to 0·39)	0·91	−0·04 (−0·47 to 0·39)	0·86
Absolute WAZ	−0·2 (−0·8 to 0·6)	−0·2 (−0·8 to 0·7)	0·00 (−0·29 to 0·30)	0·97	0·02 (−0·28 to 0·33)	0·88
Underweight (less than −2 WAZ)						
No	261/269 (97·0%)	56/57 (98·3%)	1 (Ref)	··	1 (Ref)	··
Yes	8/269 (3·0%)	1/57 (1·8%)	0·59 (0·07 to 4·72)	0·62	0·44 (0·05 to 3·83)	0·46
Height (cm)	85·0 (83·0 to 87·5)	85·0 (81·2 to 87·2)	−0·54 (−1·68 to 0·59)	0·35	−0·57 (−1·74 to 0·60)	0·34
Absolute HAZ	−0·4 (−1·2 to 0·3)	−0·2 (−1·5 to 0·4)	−0·02 (−0·39 to 0·35)	0·92	0·01 (−0·38 to 0·39)	0·98
Stunting (less than −2 HAZ)						
No	209/235 (88·9%)	47/50 (94·0%)	1 (Ref)	··	1 (Ref)	··
Yes	26/235 (11·1%)	3/50 (6·0%)	0·54 (0·16 to 1·79)	0·32	0·53 (0·16 to 1·77)	0·31
Absolute WHZ	0·2 (−0·6 to 1·0)	0·0 (−0·8 to 0·8)	−0·17 (−0·60 to 0·26)	0·44	−0·14 (−0·57 to 0·30)	0·54
Wasting (less than −2 WHZ)						
No	216/232 (93·1%)	47/50 (94·0%)	1 (Ref)	··	1 (Ref)	··
Yes	16/232 (6·9%)	3/50 (6·0%)	0·87 (0·25 to 2·99)	0·83	0·75 (0·21 to 2·60)	0·65
**30-month analysis**						
Participants attending 30-month assessment	411/553 (74·3%)	80/116 (69·0%)	··	··	··	··
Weight (kg)	12·8 (11·2 to 14·0)	12·5 (11·0 to 13·6)	−0·23 (−0·64 to 0·17)	0·25	−0·25 (−0·65 to 0·16)	0·24
Absolute WAZ	−0·1 (−0·9 to 0·7)	−0·2 (−1·2 to 0·3)	−0·18 (−0·44 to 0·08)	0·18	−0·16 (−0·43 to 0·11)	0·24
Underweight (less than −2 WAZ)						
No	368/389 (94·6%)	71/76 (93·4%)	1 (Ref)	··	1 (Ref)	··
Yes	21/389 (5·4%)	5/76 (6·6%)	1·22 (0·46 to 3·23)	0·69	1·19 (0·44 to 3·19)	0·73
Height (cm)	89·0 (86·0 to 92·0)	89·0 (86·0 to 92·8)	−0·09 (−1·08 to 0·89)	0·86	−0·09 (−1·10 to 0·92)	0·86
Absolute HAZ	−0·5 (−1·3 to 0·4)	−0·3 (−1·2 to 0·4)	0·04 (−0·24 to 0·33)	0·76	0·08 (−0·21 to 0·37)	0.60
Stunting (less than −2 HAZ)						
No	337/362 (93·1%)	70/73 (95·9%)	1 (Ref)	··	1 (Ref)	··
Yes	25/362 (6·9%)	3/73 (4·1%)	0·60 (0·18 to 1·97)	0·40	0·58 (0·17 to 1·50)	0·38
Absolute WHZ	0·3 (−0·8 to 1·1)	0·2 (−1·0 to 0·8)	−0·29 (−0·65 to 0·06)	0·11	−0·30 (−0·66 to 0·06)	0·10
Wasting (less than −2 WHZ)						
No	326/356 (91·6%)	60/73 (82·2%)	1 (Ref)	··	1 (Ref)	··
Yes	30/356 (8·4%)	13/73 (17·8%)	2·11 (1·10 to 4·05)	0·024	2·33 (1·20 to 4·52)	0·012
**36-month analysis**						
Participants attending 36-month assessment	471/553 (85·2%)	106/116 (91·4%)	··	··	··	··
Weight (kg)	14·0 (12·8 to 15·0)	14·0 (12·2 to 15·0)	0·00 (−0·37 to 0·37)	1·00	−0·15 (−0·53 to 0·24)	0·45
Absolute WAZ	−0·2 (−0·8 to 0·4)	−0·0 (−1·0 to 0·6)	−0·04 (−0·26 to 0·19)	0·76	−0·11 (−0·34 to −0·11)	0·33
Underweight (less than −2 WAZ)						
No	420/442 (95·0%)	90/95 (94·7%)	1 (Ref)	··	1 (Ref)	··
Yes	22/442 (5·0%)	5/95 (5·3%)	1·06 (0·40 to 2·79)	0·91	1·20 (0·43 to 3·32)	0·72
Height (cm)	94·0 (91·5 to 97·0)	95·0 (91·6 to 97·0)	0·07 (−0·79 to 0·94)	0·87	−0·01 (−0·89 to 0·88)	
Absolute HAZ	−0·2 (−1·0 to 0·5)	−0·2 (−1·2 to 0·5)	−0·02 (−0·26 to 0·22)	0·87	−0·02 (−0·26 to 0·22)	0·86
Stunting (less than −2 HAZ)						
No	397/415 (95·7%)	88/92 (95·7%)	1 (Ref)	··	1 (Ref)	··
Yes	18/415 (4·3%)	4/92 (4·4%)	1·00 (0·34 to 2·96)	1·00	1·04 (0·34 to 3·17)	0·95
Absolute WHZ	0·0 (−0·9 to 0·8)	0·1 (−0·8 to 0·7)	0·01 (−0·28 to 0·30)	0·96	−0·08 (−0·37 to 0·22)	0·61
Wasting (less than −2 WHZ)						
No	380/412 (92·2%)	82/90 (91·1%)	1 (Ref)	··	1 (Ref)	··
Yes	32/412 (7·8%)	8/90 (8·9%)	1·14 (0·53 to 2·48)	0·73	1·39 (0·63 to 3·10)	0·42

Data are n (%) or median (IQR) unless otherwise specified. The overall N for never PrEP exposed was 3505 and the overall N for any PrEP exposure during pregnancy was 558. HAZ=height-for-age Z score. PrEP=pre-exposure prophylaxis. WAZ=weight-for-age Z score. WHZ=weight-for-height Z score.

* Adjusted for maternal age at enrolment, primigravida at enrolment, gestational age at birth, partner HIV status at enrolment, and syphilis status at enrolment.

† Prevalence ratios are shown for binary outcomes and β-coefficients are shown for continuous outcomes.

**Table 5:** Developmental outcomes at 24 months and 36 months by prenatal PrEP exposure

	Never PrEP exposed	Any PrEP exposure during pregnancy	Unadjusted regression		Adjusted regression*	
			Coefficient† (95% CI)	p value	Coefficient† (95% CI)	p value*

**24-month analysis**						
Participants attending 24-month assessment	282/553 (51·0%)	61/116 (52·6%)	··	··	··	··
ASQ-SE score	65·0 (41·3 to 93·0)	82·7 (30·0 to 124·0)	11·94 (−21·15 to 45·04)	0·47	27·73 (−7·87 to 62·33)	0·12
Above ASQ-SE score cutoff						
No	19/37 (51·4%)	3/6 (50·0%)	1 (Ref)	··	1 (Ref)	··
Yes	18/37 (48·7%)	3/6 (50·0%)	1·00 (0·30 to 3·49)	0·97	1·55 (0·38 to 6·28)	0·54
30-month analysis						
Participants attending 30-month assessment	411/553 (74·3%)	80/116 (69·0%)	··	··	··	··
ASQ-SE score	55·0 (35·0 to 85·0)	56·9 (35·0 to 80·0)	−2·83 (−18·37 to 12·70)	0·72	−1·50 (−18·60 to 13·47)	0·75
Above ASQ-SE score cut-off						
No	108/139 (77·7%)	20/25 (80·0%)	1 (Ref)	··	1 (Ref)	··
Yes	31/139 (22·3%)	5/25 (20·0%)	0·90 (0·35 to 2·31)	0·82	0·85 (0·33 to 2·24)	0·75
**36-month analysis**						
Participants attending 36-month assessment	471/553 (85·2%)	106/116 (91·4%)	··	··	··	··
ASQ-SE score	55·0 (39·5 to 79·0)	60·0 (45·2 to 80·0)	3·26 (−5·63 to 12·15)	0·47	1·10 (−8·02 to 10·23)	0·81
Above ASQ-SE score cut-off						
No	297/347 (85·6%)	62/71 (87·3%)	1 (Ref)	··	1 (Ref)	··
Yes	50/347 (14·4%)	9/71 (12·7%)	0·88 (0·43 to 1·79)	0·72	0·82 (0·40 to 1·69)	0·60

Data are n (%) or median (IQR) unless otherwise specified. The overall N for never PrEP exposed was 3505 and the overall N for any PrEP exposure during pregnancy was 558. ASQ-SE=Ages and Stages Questionnaire – Social Emotional, 2nd edition. PrEP=pre-exposure prophylaxis.

* Adjusted for maternal age at enrolment, primigravida at enrolment, gestational age at birth, partner HIV status at enrolment, and syphilis status at enrolment.

† Prevalence ratios are shown for binary outcomes and β-coefficients are shown for continuous outcomes.

## Data Availability

Requests to access de-identified participant data and the data dictionary used in this study can be made through the corresponding author. Data will be made available subject to a written proposal and a signed data sharing agreement.

## Research in context

### Evidence before this study

HIV incidence is elevated during periods of pregnancy and postpartum in regions with high HIV prevalence. WHO recommends offering tenofovir disoproxil fumarate-based daily oral pre-exposure prophylaxis (PrEP) to pregnant and postpartum people with substantial HIV acquisition risk. We searched PubMed for original research articles published between May 14, 2014 (ie, the first Centers for Disease Control PrEP guidelines), and June 30, 2024, on perinatal and infant outcomes following maternal PrEP use. We used the search terms: “pre-exposure prophylaxis”, “PrEP”, “pregnancy”, “in utero”, “maternal child health”, and “safety”. Existing safety data of prenatal PrEP use among pregnant women are reassuring; however, the identified studies had limited follow-up beyond the perinatal period and the majority relied on maternal self-report of PrEP adherence, which might not accurately measure infant PrEP exposure. Building on previous research and examining outcomes that extend beyond infancy, including neurodevelopmental measures, in the context of confirmed maternal PrEP exposure, would contribute to a more comprehensive understanding of the safety profile of PrEP use during pregnancy.

### Added value of this study

To our knowledge, this study is the largest prospective study of PrEP use during pregnancy with the longest follow-up and one of the only safety evaluations to include confirmation of maternal PrEP use using quantifiable PrEP exposure, measured via drug concentrations in dried blood spots. Maternal PrEP use during pregnancy, regardless of trimester of PrEP initiation or duration of prenatal use, was not associated with significant differences in birth or infant outcomes throughout 36 months of life among Kenyan mother–infant pairs. Similar to previous safety data that relied on self-reported PrEP use, we found no differences in adverse perinatal outcomes among PrEP-exposed pregnancies, compared with pregnancies without PrEP use, which we confirmed with a biological measure.

### Implications of all the available evidence

Our findings suggest that oral PrEP use during pregnancy does not influence birth, growth, or social–emotional development throughout the first 36 months of life. Our results support previous data indicating safety of prenatal PrEP use as PrEP delivery scales up in this population. Our study contributes to the sparse data available on early childhood outcomes following antiretroviral exposure without the confounder of HIV exposure.
