# Supplementary material for: Prenatal exposure to HIV pre-exposure prophylaxis and birth, growth, and social–emotional developmental outcomes throughout early childhood in Kenya: a prospective cohort study
Source: Lancet Glob Health. Author manuscript; Available in PMC 2025 Apr 3. (PMC11964894; doi:10.1016/S2214-109X(24)00471-6)
Supplement: 1 [file NIHMS2061315-supplement-1.pdf]

THE LANCET
Global Health

Supplementary appendix 1

This translation in Swahili was submitted by the authors and we reproduce it as supplied. It has not been peer reviewed. *The Lancet*'s editorial processes have only been applied to the original in English, which should serve as reference for this manuscript.

Tafsiri hii katika Swahili iliwasilishwa na waandishi na tunatengeneza tena kama hutolewa. Haijapitiwa. Mchakato wa hariri wa Lancet Global Health umetumika tu kwa asili kwa Kiingereza, ambayo inapaswa kutumika kama kumbukumbu kwa muswada hii.

Supplement to: Gómez L, Kinuthia J, Abuna F, et al. Prenatal exposure to HIV pre-exposure prophylaxis and birth, growth, and social–emotional developmental outcomes throughout early childhood in Kenya: a prospective cohort study. *Lancet Glob Health* 2025; **13:** e467–78.

Kufikiwa kwa kinga dhidi ya VVU kabla ya kuambukizwa (PrEP), na matokeo ya kuzaliwa, ukuaji, na maendeleo ya kijamii na kihisia katika utoto wa mapema nchini Kenya: Utafiti wa kikundi cha matarajio

Usuli Wakati utekelezaji wa kinga dhidi ya VVU (PrEP) unavyoendelea kuongezeka miongoni mwa watu wajawazito, kupata data ya usalama kufuatia kufikiwa na PrEP kabla ya kuzaa kunasalia kuwa muhimu. Katika utafiti huu, tulilenga kutathmini uhusiano kati ya mfiduo wa PrEP kabla ya kuzaliwa na matokeo ya kuzaliwa pamoja na ukuaji wa watoto wachanga au watoto wadogo.

Mbinu Utafiti huu wa kikundi cha matarajio ulitumia data kutoka kwa utafiti wa *PrEP Implementation for Mothers in Antenatal Care* (NCT03070600). Washiriki walistahili kujumuishwa ikiwa walikuwa wajawazito kwa wakati huo, hawakuwa wakitumia PrEP, walikuwa na umri wa miaka 15 au zaidi, walipanga kusalia katika eneo la utafiti, hawakushiriki katika tafiti nyingine, na hawakuwa na VVU au kifua kikuu.Wanawake walijiandikisha wakati wa ujauzito katika kliniki 20 za afya ya uzazi na watoto magharibi mwa Kenya na kufuatiliwa kwa miezi 9 baada ya kujifungua. Wale ambao waliripoti kumeza PrEP katika ziara zozote za ujauzito walitambuliwa kama waliofikiwa na PrEP kabla ya kujifungua. Katika kundi lililongezewa muda, waliojiandikisha na watoto wao walifuatiliwa hadi miezi 36 baada ya kujifungua. Anthropometri ya watoto wachanga na maendeleo ya kihisia kwa kutumia maswali ya 'Ages and Stages' (ASQ-SE) yalitathminiwa na wauguzi wa utafiti waliofunzwa. Vipimo vya ukuaji wa mwili wa watoto wachanga na maendeleo ya kijamii na kihisia vilifanyika kwa kutumia *Ages and Stages Questionnaire* (ASQ-SE), toleo la pili, na vilifanywa na wauguzi waliopata mafunzo maalum. Miongoni mwa kikundi kidogo, tulithibitisha kufikiwa kwa PrEP kabla ya kuzaa kwa kutumia viwango vya tenofovir-diphosphate katika madoa ya damu kavu. Matokeo ya msingi yaliyotathminiwa yalihusisha kuzaliwa, ukuaji, na maendeleo ya neva.

Matokeo Kati ya Januari 15, 2018, na Julai 31, 2019, wanawake 4063 walijiandikisha na kujumuishwa katika uchanganuzi, kati yao 558 (13.7%) walitumia PrEP wakati wa ujauzito, wakianza katika wastani wa wiki 26 za ujauzito (IQR 22-31) kwa muda wa wastani wa wiki 9.6 katika ujauzito. (5.7-15.0). Ikilinganishwa na mimba ambazo hazikufikiwa na PrEP,

hakukuwa na tofauti katika kupoteza mimba, kuzaa mtoto mfu, kuzaliwa kabla ya wakati, au kifo cha mtoto mchanga kati ya mimba zizofikiwa na PrEP (zote $p>0.05$). Hakukuwa na utofauti katika urefu au uzito wa watoto wachanga katika wiki 6, miezi 6na miezi 9 (zote $p>0.05$) kati ya wasio na waliofikiwa na PrEP kabla ya kuzaa, ikiwa ni pamoja na uzito mdogo, kudumaa, na kudhoofika. Matokeo yalikuwa sawa yalipochanganuliwa kando kuangazia muda wa kuanza PrEP na muda waliotumia PrEP na katika kikundi kidogo miezi 24, miezi 30 na miezi 36. Kufikiwa kwa kabla ya kuzaa na PrEP haukuhusishwa na alama za ASQ-SE katika miezi 24 ($p=0.12$), miezi 30 ($p=0.75$), au miezi 36 ($p=0.81$). Hakuna tofauti katika matokeo mabaya ya uzazi na kwa watoto wachanga yaliyopatikana miongoni mwa washiriki wa Kenya waliofikiwa na viwango vilivyoweza kupimwa vya TFV-DP kabla ya kuzaa.

Ufafanuzi Hatukupata tofauti ya maana katika kuzaa au matokeo mabaya ya uzazi kwa muda wa miaka 3 ya ufuatiliaji wa hali ya kufikiwa na PrEP kabla ya kuzaa. Data hizi zinaunga mkono matokeo ya tafiti za awali zinazoonyesha usalama wa matumizi ya PrEP ya kumezwa wakati wa ujauzito
