# Supplementary material for: Prenatal exposure to HIV pre-exposure prophylaxis and birth, growth, and social–emotional developmental outcomes throughout early childhood in Kenya: a prospective cohort study
Source: Lancet Glob Health. Author manuscript; Available in PMC 2025 Apr 3. (PMC11964894; doi:10.1016/S2214-109X(24)00471-6)
Supplement: 2 [file NIHMS2061315-supplement-2.pdf]

THE LANCET
Global Health

Supplementary appendix 2

This appendix formed part of the original submission and has been peer reviewed. We post it as supplied by the authors.

Supplement to: Gómez L, Kinuthia J, Abuna F, et al. Prenatal exposure to HIV pre-exposure prophylaxis and birth, growth, and social–emotional developmental outcomes throughout early childhood in Kenya: a prospective cohort study. *Lancet Glob Health* 2025; **13:** e467–78.

Supplemental Figure 1· Flow diagram of inclusion

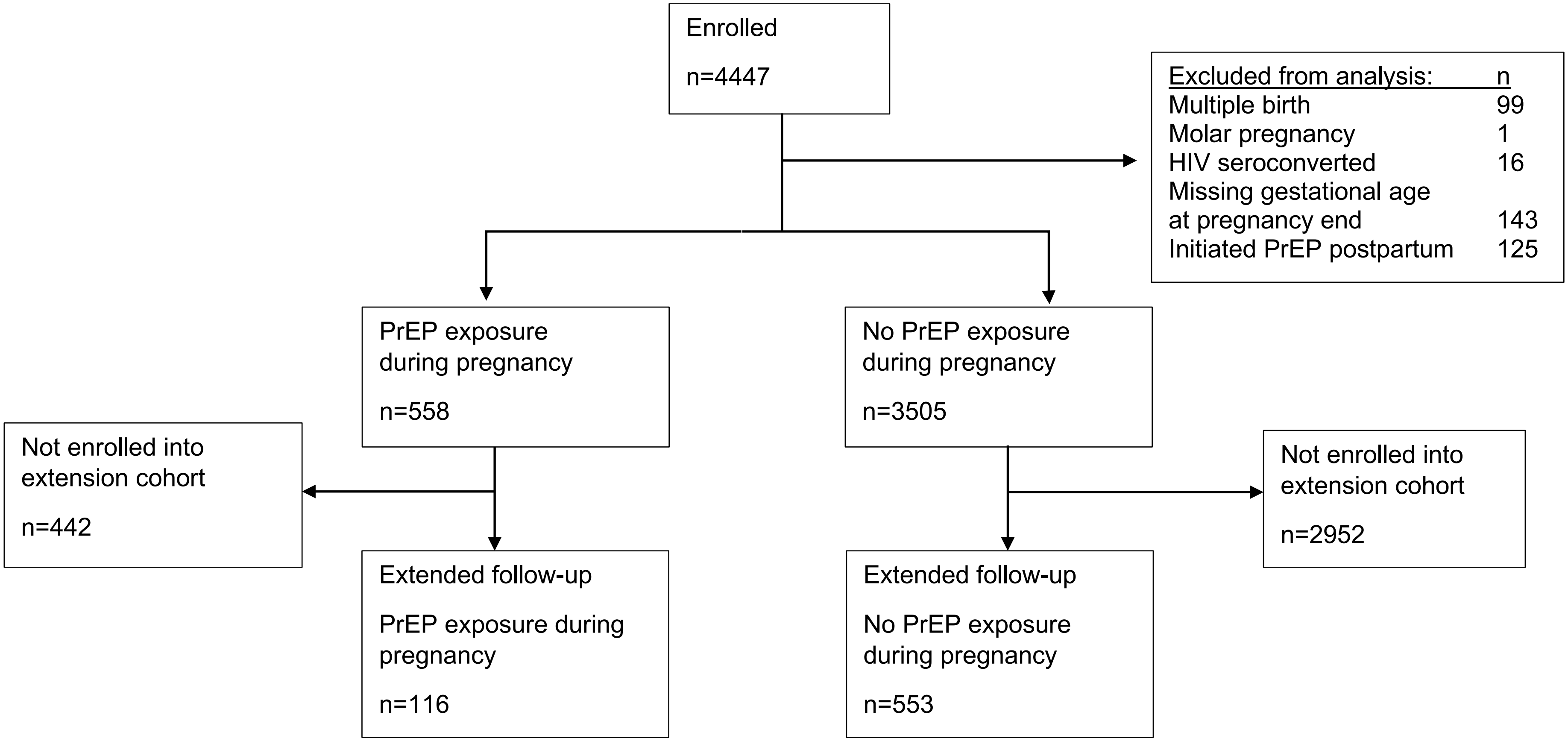

Supplemental Table 1· Perinatal outcomes by trimester of first PrEP exposure and duration of PrEP exposure during pregnancy

	Gestational Age at Pregnancy End (weeks)				Pre-term Birth (<37 weeks gestation)			
	N	Median (IQR)	Coefficient[1] (95% CI)	*P*[2]	N	n (%)	Coefficient[1] (95% CI)	*P*[2]
**Trimester of first PrEP exposure**								
No PrEP exposure	3444	38·0 (37·0, 39·0)	*Ref·*		3444	661 (19·2)	*Ref·*	
1st trimester	56	38·0 (37·0, 40·0)	-0·24 (-1·73, 1·25)	0·740	56	11 (19·6)	0·96 (0·50, 1·83)	0·903
2nd trimester	286	38·0 (37·0, 39·0)	-0·13 (-0·51, 0·26)	0·502	286	63 (22·0)	1·20 (0·92, 1·56)	0·185
3rd trimester	213	38·0 (38·0, 39·0)	0·18 (-0·13, 0·50)	0·237	213	20 (9·4)	0·57 (0·40, 0·82)	0·002
**Duration of PrEP exposure during pregnancy**								
No PrEP exposure	3444	38·0 (37·0, 39·0)	*Ref·*		3444	661 (19·2)	*Ref·*	
<4wks	47	38·0 (37·0, 39·0)	-0·76 (-1·74, 0·22)	0·121	47	8 (17·0)	1·07 (0·59, 1·94)	0·826
4-12wks	291	38·0 (37·0, 39·0)	-0·26 (-0·64, 0·12)	0·164	291	51 (17·5)	1·02 (0·75, 1·39)	0·910
>12wks	217	38·0 (38·0, 40·0)	0·49 (0·15, 0·82)	0·006	217	35 (16·1)	0·84 (0·56, 1·27)	0·411

	Congenital Malformation				Neonatal Death			
	N	n (%)	Coefficient[1] (95% CI)	*P*[2]	N	n (%)	Coefficient[1] (95% CI)	*P*[2]
**Trimester of PrEP initiation**								
Unexposed	3430	16 (0·5)	*Ref·*		3430	55 (1·6)	*Ref·*	
1st trimester	53	1 (1·9)	6·63 (1·35, 32·49)	0·020	53	0 (0·0)	-	-
2nd trimester	275	2 (0·7)	1·92 (0·48, 7·66)	0·354	275	5 (1·8)	1·36 (0·38, 4·92)	0·640
3rd trimester	213	2 (0·9)	2·00 (0·43, 9·39)	0·378	213	5 (2·3)	1·31 (0·54, 3·17)	0·544
**Duration of PrEP exposure during pregnancy**								
Unexposed	3430	16 (0·5)	*Ref·*		3430	55 (1·6)	*Ref·*	
<4wks	48	0 (0·0)	-	-	48	3 (6·3)	3·48 (0·99, 12·24)	0·052

4-12wks	280	1 (0·4)	0·87 (0·10, 7·73)	0·897	280	6 (2·1)	1·38 (0·65, 2·90)	0·398
>12wks	213	4 (1·9)	5·29 (2·06, 13·62)	0·001	213	1 (0·5)	0·35 (0·05, 2·36)	0·284
	**Loss of pregnancy (miscarriage, <20 weeks gestation)**				**Stillbirth (≥20 weeks gestation)**			
	**N**	**n (%)**	**Coefficient[1] (95% CI)**	***P*[2]**	**N**	**n (%)**	**Coefficient[1] (95% CI)**	***P*[2]**
**Trimester of first PrEP exposure**								
No PrEP exposure	841	10 (1·2)	*Ref·*		841	26 (3·1)	*Ref·*	
1st trimester	56	2 (3·6)	2·61 (0·38, 17·96)	0·329	56	1 (1·8)	0·59 (0·09, 3·64)	0·566
2nd trimester	97	0 (0·0)	-	-	97	4 (4·1)	0·89 (0·27, 2·92)	0·842
3rd trimester	3	0 (0·0)	-	-	3	0 (0·0)	-	-
**Duration of PrEP exposure during pregnancy**								
No PrEP exposure	841	10 (1·2)	*Ref·*		841	26 (3·1)	*Ref·*	
<4wks	3	0 (0·0)	-	-	3	0 (0·0)	-	-
4-12wks	48	2 (4·2)	5·01 (0·91, 27·62)	0·064	48	3 (6·3)	2·04 (0·71, 5·89)	0·187
>12wks	105	0 (0·0)	-	-	105	2 (1·9)	0·27 (0·03, 2·14)	0·216
	**Birthweight (kilograms)**				**Low Birth Weight (<2·5 kilograms)**			
	**N**	**Median (IQR)**	**Coefficient[1] (95% CI)**	***P*[2]**	**N**	**n (%)**	**Coefficient[1] (95% CI)**	***P*[2]**
**Trimester of first PrEP exposure**								
No PrEP exposure	2160	3·4 (3·0, 3·6)	*Ref·*		2160	50 (2·3)	*Ref·*	
1st trimester	28	3·3 (3·0, 3·7)	-0·16 (-0·35, 0·03)	0·098	28	1 (3·6)	1·27 (0·17, 9·68)	0·820
2nd trimester	210	3·4 (3·0, 3·6)	-0·04 (-0·12, 0·04)	0·293	210	3 (1·4)	0·61 (0·23, 1·63)	0·321
3rd trimester	152	3·3 (3·0, 3·6)	-0·01 (-0·11, 0·09)	0·851	152	2 (1·3)	0·65 (0·15, 2·88)	0·574
**Duration of PrEP exposure during pregnancy**								
No PrEP exposure	2160	3·4 (3·0, 3·6)	*Ref·*		2160	50 (2·3)	*Ref·*	

<4wks	36	3·3 (3·2, 3·5)	-0·04 (-0·26, 0·17)	0·697	36	1 (2·8)	1·41 (0·19, 10·51)	0·741
4-12wks	205	3·2 (3·0, 3·5)	-0·05 (-0·11, 0·00)	**0·062**	205	3 (1·5)	0·68 (0·27, 1·74)	0·424
>12wks	149	3·4 (3·0, 3·7)	-0·01 (-0·12, 0·09)	0·804	149	2 (1·3)	0·53 (0·17, 1·69)	0·286
	**Small for Gestational Age regardless of sex**				**Small for Gestational Age separately by sex**			
	**N**	**Median (IQR)**	**Coefficient[1] (95% CI)**	***P*[2]**	**N**	**n (%)**	**Coefficient[1] (95% CI)**	***P*[2]**
**Trimester of first PrEP exposure**								
No PrEP exposure	2160	230 (10·6)	*Ref·*		2156	212 (9·8)	*Ref·*	
1st trimester	28	6 (21·4)	2·88 (1·53, 5·42)	0·001	28	6 (21·4)	2·80 (1·46, 5·39)	0·002
2nd trimester	210	24 (11·4)	1·20 (0·88, 1·62)	0·245	210	21 (10·0)	1·10 (0·76, 1·58)	0·621
3rd trimester	152	16 (10·5)	0·87 (0·51, 1·48)	0·606	152	15 (9·9)	0·89 (0·50, 1·61)	0·708
Duration of PrEP exposure during pregnancy								
No PrEP exposure	2160	230 (10·6)	*Ref·*		2156	212 (9·8)	*Ref·*	
<4wks	36	1 (2·8)	0·25 (0·03, 1·81)	0·171	36	1 (2·8)	0·28 (0·04, 2·08)	0·213
4-12wks	205	28 (13·7)	1·23 (0·82, 1·85)	0·323	205	26 (12·7)	1·22 (0·76, 1·96)	0·401
>12wks	149	17 (11·4)	1·24 (0·81, 1·90)	0·327	149	15 (10·1)	1·13 (0·70, 1·83)	0·612

PrEP=pre-exposure prophylaxis. P=P-value.
[1]Generalised estimating equations regression. Prevalence ratios shown for binary outcomes and β-coefficients shown for continuous outcomes
[2]Adjusted for maternal age at enrolment, primigravida at enrolment, gestational age at enrolment, partner HIV status at enrolment, and syphilis status at enrolment

Supplemental Table 2· Infant growth outcomes at 6-weeks, 6-months, and 9-months postpartum by trimester of PrEP initiation and duration of PrEP exposure during pregnancy

	**6-Week Absolute WAZ**				**6-Week Underweight (<-2 WAZ)**			
	**N**	**Median (IQR)**	**Coefficient[1] (95% CI)**	***P*[2]**	**N**	**n (%)**	**Coefficient[1] (95% CI)**	***P*[2]**
**Trimester of PrEP initiation**								
Unexposed	2151	0·3 (-0·4, 0·9)	*Ref·*		2151	65 (3·0)	*Ref·*	

1st trimester	37	0·1 (-0·6, 1·0)	-0·13 (-0·49, 0·24)	0·482	37	1 (2·7)	1·36 (0·18, 10·07)	0·766
2nd trimester	157	0·4 (-0·5, 0·8)	-0·07 (-0·22, 0·09)	0·403	157	5 (3·2)	1·16 (0·58, 2·31)	0·674
3rd trimester	97	0·2 (-0·3, 0·7)	0·05 (-0·19, 0·28)	0·688	97	1 (1·0)	0·32 (0·04, 2·28)	0·254
**Duration of PrEP exposure during pregnancy**								
Unexposed	2151	0·3 (-0·4, 0·9)	*Ref·*		2151	65 (3·0)	*Ref·*	
<4wks	18	-0·2 (-0·9, 0·5)	-0·51 (-1·22, 0·20)	0·149	18	1 (5·6)	1·78 (0·23, 13·69)	0·581
4-12wks	139	0·2 (-0·5, 0·7)	-0·02 (-0·20, 0·16)	0·828	139	3 (2·2)	0·72 (0·23, 2·23)	0·571
>12wks	134	0·4 (-0·4, 0·9)	0·01 (-0·17, 0·20)	0·874	134	3 (2·2)	0·83 (0·34, 2·06)	0·696
	**6-Week Absolute LAZ**				**6-Week Stunting (<-2 LAZ)**			
	**N**	**Median (IQR)**	**Coefficient[1] (95% CI)**	***P*[2]**	**N**	**n (%)**	**Coefficient[1] (95% CI)**	***P*[2]**
**Trimester of PrEP initiation**								
Unexposed	2108	-0·2 (-1·1, 0·7)	*Ref·*		2108	201 (9·5)	*Ref·*	
1st trimester	37	-0·2 (-0·9, 0·7)	0·14 (-0·42, 0·70)	0·603	37	4 (10·8)	1·29 (0·47, 3·58)	0·620
2nd trimester	156	-0·1 (-0·8, 0·6)	0·04 (-0·21, 0·29)	0·719	156	16 (10·3)	1·19 (0·74, 1·92)	0·465
3rd trimester	93	-0·2 (-1·0, 0·8)	0·00 (-0·32, 0·32)	0·997	93	9 (9·7)	1·19 (0·51, 2·78)	0·688
**Duration of PrEP exposure during pregnancy**								
Unexposed	2108	-0·2 (-1·1, 0·7)	*Ref·*		2108	201 (9·5)	*Ref·*	
<4wks	16	-0·8 (-2·1, -0·1)	-1·03 (-1·96, -0·11)	0·030	16	4 (25·0)	3·00 (0·98, 9·19)	0·054
4-12wks	138	-0·2 (-1·0, 0·9)	0·07 (-0·21, 0·35)	0·597	138	13 (9·4)	1·09 (0·74, 1·60)	0·666
>12wks	132	-0·1 (-0·8, 0·6)	0·14 (-0·16, 0·43)	0·343	132	12 (9·1)	1·11 (0·56, 2·19)	0·770
	**6-Week Absolute WLZ**				**6-Week Wasting (<-2 WLZ)**			
	**N**	**Median (IQR)**	**Coefficient[1] (95% CI)**	***P*[2]**	**N**	**n (%)**	**Coefficient[1] (95% CI)**	***P*[2]**
**Trimester of PrEP initiation**								

Unexposed	2070	0·6 (-0·3, 1·7)	*Ref·*		2070	121 (5·8)	*Ref·*	
1st trimester	37	0·6 (0·0, 1·4)	-0·24 (-0·75, 0·27)	0·333	37	1 (2·7)	0·57 (0·08, 4·13)	0·574
2nd trimester	150	0·4 (-0·4, 1·5)	-0·11 (-0·39, 0·17)	0·429	150	8 (5·3)	0·94 (0·46, 1·93)	0·876
3rd trimester	93	0·8 (-0·1, 1·8)	0·22 (-0·15, 0·60)	0·227	93	5 (5·4)	0·70 (0·22, 2·28)	0·555
**Duration of PrEP exposure during pregnancy**								
Unexposed	2070	0·6 (-0·3, 1·7)	*Ref·*		2070	121 (5·8)	*Ref·*	
<4wks	17	0·8 (0·3, 1·8)	0·50 (-0·09, 1·10)	0·092	17	0 (0·0)	-	-
4-12wks	132	0·5 (-0·3, 1·6)	-0·01 (-0·35, 0·34)	0·966	132	8 (6·1)	0·91 (0·46, 1·81)	0·787
>12wks	131	0·6 (-0·3, 1·5)	-0·08 (-0·33, 0·16)	0·490	131	6 (4·6)	0·83 (0·30, 2·26)	0·711
	**6-Month Absolute WAZ**				**6-Month Underweight (<-2 WAZ)**			
	**N**	**Median (IQR)**	**Coefficient[1] (95% CI)**	***P*[2]**	**N**	**n (%)**	**Coefficient[1] (95% CI)**	***P*[2]**
**Trimester of PrEP initiation**								
Unexposed	1737	0·1 (-0·7, 0·9)	*Ref·*		1737	59 (3·4)	*Ref·*	
1st trimester	31	0·3 (-0·5, 1·2)	0·24 (-0·24, 0·72)	0·308	31	0 (0·0)	-	-
2nd trimester	137	0·2 (-0·4, 1·2)	0·21 (-0·01, 0·44)	0·064	137	3 (2·4)	0·61 (0·17, 2·14)	0·438
3rd trimester	87	0·2 (-0·4, 0·9)	0·17 (-0·08, 0·41)	0·172	87	3 (3·4)	0·90 (0·26, 3·08)	0·868
**Duration of PrEP exposure during pregnancy**								
Unexposed	1737	0·1 (-0·7, 0·9)	*Ref·*		1737	59 (3·4)	*Ref·*	
<4wks	14	-0·1 (-1·1, 0·6)	-0·29 (-0·84, 0·25)	0·271	14	0 (0·0)	-	-
4-12wks	127	0·3 (-0·4, 1·1)	0·26 (0·03, 0·49)	0·030	127	4 (3·1)	0·84 (0·31, 2·26)	0·728
>12wks	114	0·3 (-0·4, 1·2)	0·20 (-0·03, 0·42)	**0·082**	114	2 (1·8)	0·51 (0·07, 3·70)	0·506
	**6-Month Absolute LAZ**				**6-Month Stunting (<-2 LAZ)**			
	**N**	**Median (IQR)**	**Coefficient[1] (95% CI)**	***P*[2]**	**N**	**n (%)**	**Coefficient[1] (95% CI)**	***P*[2]**

**Trimester of PrEP initiation**								
Unexposed	1725	-0·3 (-1·1, 0·6)	*Ref·*		1725	150 (8·7)	*Ref·*	
1st trimester	32	-0·2 (-1·2, 0·9)	-0·01 (-0·80, 0·77)	0·975	32	4 (12·5)	2·12 (0·89, 5·05)	0·091
2nd trimester	134	-0·4 (-1·1, 0·8)	-0·08 (-0·37, 0·22)	0·588	134	12 (9·0)	1·19 (0·74, 1·91)	0·475
3rd trimester	88	-0·0 (-1·0, 0·8)	0·22 (-0·03, 0·47)	**0·076**	88	5 (5·7)	0·63 (0·29, 1·39)	0·253
**Duration of PrEP exposure during pregnancy**								
Unexposed	1725	-0·3 (-1·1, 0·6)	*Ref·*		1725	150 (8·7)	*Ref·*	
<4wks	14	-0·2 (-1·0, 0·3)	0·04 (-0·30, 0·39)	0·795	14	0 (0·0)	-	-
4-12wks	129	-0·3 (-1·1, 0·6)	-0·04 (-0·30, 0·23)	0·772	129	11 (8·5)	1·01 (0·55, 1·85)	0·976
>12wks	111	-0·1 (-0·9, 1·1)	0·13 (-0·31, 0·57)	0·552	111	10 (9·0)	1·27 (0·79, 2·04)	0·320
	**6-Month Absolute WLZ**				**6-Month Wasting (<-2 WLZ)**			
	**N**	**Median (IQR)**	**Coefficient[1] (95% CI)**	***P*[2]**	**N**	**n (%)**	**Coefficient[1] (95% CI)**	***P*[2]**
**Trimester of PrEP initiation**								
Unexposed	1712	0·4 (-0·5, 1·4)	*Ref·*		1712	67 (3·9)	*Ref·*	
1st trimester	31	0·4 (-0·8, 2·1)	0·25 (-0·41, 0·90)	0·439	31	1 (3·2)	0·83 (0·09, 7·44)	0·869
2nd trimester	131	0·7 (-0·3, 1·6)	0·23 (-0·05, 0·50)	0·098	131	3 (2·3)	0·55 (0·21, 1·45)	0·227
3rd trimester	86	0·3 (-0·5, 1·4)	0·02 (-0·23, 0·28)	0·843	86	3 (3·5)	0·75 (0·24, 2·41)	0·632
**Duration of PrEP exposure during pregnancy**								
Unexposed	1712	0·4 (-0·5, 1·4)	*Ref·*		1712	67 (3·9)	*Ref·*	
<4wks	14	-0·1 (-0·8, 0·9)	-0·47 (-1·34, 0·41)	0·277	14	2 (14·3)	3·15 (0·61, 16·19)	**0·170**
4-12wks	126	0·5 (-0·3, 1·8)	0·36 (0·10, 0·62)	0·009	126	1 (0·8)	0·18 (0·03, 1·26)	0·084
>12wks	108	0·5 (-0·5, 1·3)	0·00 (-0·28, 0·29)	0·990	108	4 (3·7)	0·89 (0·35, 2·26)	0·807
	**9-Month Absolute WAZ**				**9-Month Underweight (<-2 WAZ)**			

	N	Median (IQR)	Coefficient[1] (95% CI)	*P*[2]	N	n (%)	Coefficient[1] (95% CI)	*P*[2]
**Trimester of PrEP initiation**								
Unexposed	1738	0·1 (-0·7, 1·0)	*Ref·*		1738	67 (3·9)	*Ref·*	
1st trimester	31	0·2 (-0·8, 1·0)	-0·04 (-0·46, 0·39)	0·863	31	1 (3·2)	1·11 (0·17, 7·33)	0·917
2nd trimester	150	0·1 (-0·5, 0·9)	0·04 (-0·12, 0·19)	0·632	150	3 (2·0)	0·55 (0·21, 1·42)	0·215
3rd trimester	95	0·1 (-0·6, 0·7)	0·08 (-0·16, 0·31)	0·493	95	4 (4·2)	0·95 (0·34, 2·66)	0·916
**Duration of PrEP exposure during pregnancy**								
Unexposed	1738	0·1 (-0·7, 1·0)	*Ref·*		1738	67 (3·9)	*Ref·*	
<4wks	22	0·1 (-0·5, 0·5)	-0·23 (-0·73, 0·28)	0·363	22	2 (9·1)	2·15 (0·55, 8·38)	0·271
4-12wks	137	0·2 (-0·5, 1·0)	0·15 (-0·05, 0·34)	0·128	137	3 (2·2)	0·54 (0·17, 1·74)	0·303
>12wks	117	0·0 (-0·6, 0·8)	-0·03 (-0·21, 0·15)	0·734	117	3 (2·6)	0·72 (0·20, 2·61)	0·614
	**9-Month Absolute LAZ**				**9-Month Stunting (<-2 LAZ)**			
	**N**	**Median (IQR)**	**Coefficient[1] (95% CI)**	***P*[2]**	**N**	**n (%)**	**Coefficient[1] (95% CI)**	***P*[2]**
**Trimester of PrEP initiation**								
Unexposed	1701	-0·4 (-1·3, 0·5)	*Ref·*		1701	157 (9·2)	*Ref·*	
1st trimester	31	-0·2 (-1·1, 0·8)	0·24 (-0·27, 0·76)	0·334	31	0 (0·0)	-	-
2nd trimester	144	-0·1 (-0·9, 0·6)	0·12 (-0·15, 0·38)	0·378	144	10 (6·9)	0·85 (0·47, 1·54)	0·590
3rd trimester	92	-0·5 (-1·3, 0·4)	-0·06 (-0·40, 0·29)	0·730	92	8 (8·7)	0·94 (0·47, 1·88)	0·854
**Duration of PrEP exposure during pregnancy**								
Unexposed	1701	-0·4 (-1·3, 0·5)	*Ref·*		1701	157 (9·2)	*Ref·*	
<4wks	21	-1·0 (-1·5, -0·1)	-0·65 (-1·29, -0·01)	0·048	21	3 (14·3)	1·66 (0·58, 4·77)	0·344
4-12wks	134	-0·3 (-1·0, 0·5)	0·02 (-0·24, 0·28)	0·872	134	10 (7·5)	0·85 (0·42, 1·73)	0·658
>12wks	112	-0·0 (-1·0, 0·7)	0·27 (-0·06, 0·60)	0·105	112	5 (4·5)	0·56 (0·23, 1·38)	0·208

	9-Month Absolute WLZ				9-Month Wasting (<-2 WLZ)			
	**N**	**Median (IQR)**	**Coefficient[1] (95% CI)**	***P*[2]**	**N**	**n (%)**	**Coefficient[1] (95% CI)**	***P*[2]**
**Trimester of PrEP initiation**								
Unexposed	1691	0·4 (-0·5, 1·3)	*Ref·*		1691	60 (3·5)	*Ref·*	
1st trimester	31	0·4 (-0·8, 1·3)	-0·13 (-0·70, 0·44)	0·629	31	1 (3·2)	0·73 (0·13, 3·97)	0·718
2nd trimester	144	0·4 (-0·5, 1·3)	-0·01 (-0·28, 0·26)	0·923	144	4 (2·8)	0·71 (0·27, 1·84)	0·477
3rd trimester	92	0·5 (-0·4, 1·4)	0·15 (-0·12, 0·42)	0·259	92	3 (3·3)	0·94 (0·27, 3·32)	0·921
**Duration of PrEP exposure during pregnancy**								
Unexposed	1691	0·4 (-0·5, 1·3)	*Ref·*		1691	60 (3·5)	*Ref·*	
<4wks	21	0·7 (0·1, 1·3)	0·21 (-0·36, 0·77)	0·455	21	1 (4·8)	1·44 (0·17, 11·97)	0·736
4-12wks	134	0·5 (-0·3, 1·5)	0·20 (-0·08, 0·48)	0·147	134	3 (2·2)	0·61 (0·17, 2·11)	0·431
>12wks	112	0·0 (-0·6, 1·1)	-0·22 (-0·49, 0·06)	0·116	112	4 (3·6)	0·89 (0·31, 2·51)	0·818

The overall N for never PrEP exposed was 3505 and the overall N for any PrEP exposure during pregnancy was 558.
PrEP=pre-exposure prophylaxis. P=p-value. LAZ=length-for-age Z score. WAZ=weight-for-age Z score. WLZ=weight-for-length Z score.
[1] Generalised estimating equations regression. Prevalence ratios shown for binary outcomes and β-coefficients shown for continuous outcomes
[2] Adjusted for maternal age at enrolment, primigravida at enrolment, gestational age at enrolment, partner HIV status at enrolment, and syphilis status at enrolment

Supplemental Table 3· Perinatal outcomes by confirmed prenatal PrEP exposure

		Median (IQR) or n (%)		Unadjusted GEE regression		Adjusted GEE regression[1]	
		Any quantifiable PrEP exposure during pregnancy					
	**N (n=3603)**	**No (n=3505)**	**Yes (n=103)**	**Coeff[2](95% CI)**	***P***	**Coeff[2] (95% CI)**	***P***
Gestational age at pregnancy end	3547	38·0 (37·0, 39·0)	38·0 (38·0, 40·0)	0·35 (0·02, 0·68)	0·041	0·31 (-0·06, 0·68)	0·098
Preterm birth (<37 weeks gestation)	3547						
No	2870	2783 (80·8)	87 (84·5)	ref		ref	
Yes	677	661 (19·2)	16 (15·5)	0·81 (0·51, 1·30)	0·379	0·92 (0·58, 1·47)	0·729

Loss of pregnancy (<20 weeks)[2]	867						
No	857	831 (98·8)	26 (100)	ref		ref	
Yes	10	10 (1·2)	0 (10)	-	-	-	-
Stillbirth (≥20 weeks gestation)	867						
No	840	815 (96·9)	25 (96·2)	ref		ref	
Yes	27	26 (3·1)	1 (3·8)	1·24 (0·14, 11·36)	0·847	1·03 (0·12, 8·93)	0·977
Birthweight (kilograms)	2235	3·4 (3·0, 3·6)	3·2 (3·0, 3·5)	-0·14 (-0·25, -0·02)	0·024	-0·16 (-0·28, -0·04)	0·012
Birthweight (<2·5 kilograms)[2]	2235						
No	2185	2110 (97·7)	75 (100)	ref		ref	
Yes	50	50 (2·3)	0 (0)	-	-	-	-
Small for gestational age	2231						
No	2009	1944 (90·2)	65 (86·7)	ref		ref	
Yes	222	212 (9·8)	10 (13·3)	1·36 (0·76, 2·43)	0·307	1·41 (0·78, 2·54)	0·259
Congenital malformation[2]	3530						
No	3514	3414 (99·5)	100 (100·0)	ref		ref	
Yes	16	16 (0·5)	0 (0·0)	-	-	-	-
Neonatal death	3530						
No	3474	3375 (98·4)	99 (99·0)	ref		ref	
Yes	56	55 (1·6)	1 (1·0)	0·62 (0·08, 4·63)	0·644	0·69 (0·09, 5·26)	0·718

GEE=Generalised estimating equations. Coeff=Coefficient. PrEP=pre-exposure prophylaxis

[1]Adjusted for maternal age at enrolment, primigravida at enrolment, gestational age at enrolment, partner HIV status at enrolment, and syphilis status at enrolment

[2] Prevalence ratios shown for binary outcomes and β-coefficients shown for continuous outcomes. PR and P-value not shown due to 0% prevalence among PrEP-exposed

Supplemental Table 4· Infant growth outcomes at 6-weeks, 6-months, and 9-months postpartum by confirmed PrEP exposure

		Median (IQR) or n (%)		Unadjusted GEE regression		Adjusted GEE regression[1]	
		Any quantifiable PrEP exposure during pregnancy					
	N (n=3608)	No (n=3505)	Yes (n=103)	Coefficient[2] (95% CI)	*p*	Coefficient[2] (95% CI)	*p*
**6-Weeks**	2606	2264	82				
Weight (kg)	2242	5·0 (4·5, 5·4)	5·1 (4·5, 5·6)	0·06 (-0·08, 0·20)	0·384	0·03 (-0·13, 0·19)	0·692
Absolute WAZ	2211	0·3 (-0·4, 0·9)	0·4 (-0·4, 1·1)	0·18 (-0·03, 0·38)	**0·083**	0·13 (-0·09, 0·36)	0·229
Underweight (<-2 WAZ)[2]	2211						
No	2146	2086 (97·1)	60 (100·0)	ref		ref	
Yes	65	65 (2·9)	0 (0·0)	-	-	-	-
Length (cm)	2210	55·0 (54·0, 57·0)	56·0 (54·0, 57·6)	-0·16 (-1·71, 1·39)	0·829	-0·20 (-1·78, 1·37)	0·790
Absolute LAZ	2166	-0·2 (-1·1, 0·7)	0·0 (-0·7, 0·9)	0·35 (-0·003, 0·70)	0·052	0·31 (-0·04, 0·66)	0·081
Stunting (<-2 LAZ)	2166						
No	1960	1907 (90·5)	53 (91·4)	ref		ref	
Yes	206	201 (9·5)	5 (8·6)	0·90 (0·40, 2·07)	0·811	1·15 (0·47, 2·79)	0·762
Absolute WLZ	2127	0·6 (-0·3, 1·7)	0·5 (-0·4, 1·6)	-0·19 (-0·45, 0·07)	0·138	-0·17 (-0·42, 0·07)	0·151
Wasting (<-2 WLZ)	2127						
No	2002	1949 (94·2)	53 (93·0)	ref		ref	
Yes	125	121 (5·8)	4 (7·0)	1·20 (0·47, 3·06)	0·702	1·13 (0·44, 2·93)	0·794
**6-Months**	2012	1945	67				
Weight (kg)	1886	7·7 (7·0, 8·5)	7·6 (7·1, 8·5)	0·007 (-0·35, 0·36)	0·968	0·004 (-0·38, 0·39)	0·985
Absolute WAZ	1793	0·1 (-0·7, 0·9)	-0·1 (-0·6, 0·8)	-0·03 (-0·36, 0·30)	0·846	-0·02 (-0·40, 0·35)	0·900
Underweight (<-2 WAZ)	1793						
No	1733	1678 (96·6)	55 (98·2)	ref		ref	
Yes	60	59 (3·4)	1 (1·8)	0·53 (0·09, 3·24)	0·488	0·48 (0·07, 3·22)	0·453
Length (cm)	1892	66·0 (64·0, 68·0)	66·0 (64·0, 68·8)	0·31 (-0·50, 1·12)	0·436	0·20 (-0·79, 1·19)	0·677
Absolute LAZ	1781	-0·3 (-1·1, 0·6)	-0·1 (-1·1, 0·6)	-0·10 (-0·47, 0·28)	0·600	-0·14 (-0·53, 0·25)	0·469
Stunting (<-2 LAZ)	1781						
No	1624	1575 (91·3)	49 (87·5)	ref		ref	
Yes	157	150 (8·7)	7 (12·5)	1·44 (0·66, 3·13)	0·362	1·63 (0·81, 3·27)	0·170
Absolute WLZ	1766	0·4 (-0·5, 1·4)	0·1 (-0·5, 1·3)	-0·03 (-0·33, 0·27)	0·858	0·02 (-0·31, 0·34)	0·912
Wasting (<-2 WLZ)	1766						

No	1699	1645 (96·1)	54 (100·0)	ref		ref	
Yes	67	67 (3·9)	0 (0·0)	-	-	-	-
**9-Months**	1979	1908	71				
Weight (kg)	1909	8·6 (7·9, 9·6)	8·6 (7·9, 9·5)	0·05 (-0·24, 0·33)	0·746	0·06 (-0·22, 0·34)	0·642
Absolute WAZ	1801	0·1 (-0·7, 1·0)	-0·1 (-0·5, 0·8)	0·0002 (-0·23, 0·23)	0·998	-0·01 (-0·22, 0·20)	0·895
Underweight (<-2 WAZ)	1801						
No	1732	1671 (96·1)	61 (96·8)	ref		ref	
Yes	69	67 (3·9)	2 (3·2)	0·82 (0·24, 2·85)	0·759	0·77 (0·23, 2·61)	0·676
Length (cm)	1883	70·0 (68·0, 72·0)	71·0 (69·0, 72·0)	0·97 (-0·01, 1·94)	0·052	0·70 (-0·45, 1·85)	0·219
Absolute LAZ	1762	-0·4 (-1·2, 0·5)	-0·2 (-1·0, 0·7)	0·16 (-0·17, 0·50)	0·317	0·09 (-0·25, 0·42)	0·598
Stunting (<-2 LAZ)	1762						
No	1603	1544 (90·8)	59 (93·7)	ref		ref	
Yes	159	157 (9·2)	2 (3·3)	0·36 (0·05, 2·55)	0·303	0·42 (0·06, 3·06)	0·391
Absolute WLZ	1752	0·4 (-0·5, 1·3)	0·3 (-0·6, 1·1)	-0·16 (-0·45, 0·13)	0·266	-0·10 (-0·41, 0·21)	0·510
Wasting (<-2 WLZ)	1752						
No	1689	1631 (96·5)	58 (95·1)	ref		ref	
Yes	63	60 (3·5)	3 (4·9)	1·39 (0·50, 3·86)	0·532	1·16 (0·39, 3·52)	0·788

GEE= generalised estimating equations. PrEP=pre-exposure prophylaxis. P=p-value. LAZ=length-for-age Z score. WAZ=weight-for-age Z score. WLZ=weight-for-length Z score.

[1]Adjusted for maternal age at enrollment, primigravida at enrollment, gestational age at enrollment, partner HIV status at enrollment, syphilis status at enrollment

[2]Prevalence ratios shown for binary outcomes and β-coefficients shown for continuous outcomes. PR and P-value not shown due to 0% prevalence among PrEP-exposed

Supplemental Table 5· Perinatal outcomes by confirmed prenatal PrEP exposure consistent with ≥ 2 doses/week

		Median (IQR) or n (%)		Unadjusted GEE regression		Adjusted GEE regression[2]	
		Quantified PrEP exposure ≥2 doses/week during pregnancy[1]					
	N (n=3516)	No (n=3444)	Yes (n=72)	Coefficient[3] (95% CI)	*p*	Coefficient[3] (95% CI)	*p*
Gestational age at pregnancy end	3516	38·0 (37·0, 39·0)	38·0 (38·0, 40·0)	0·41 (-0·01, 0·83)	0·056	0·37 (-0·09, 0·84)	0·109
Preterm birth (<37 weeks gestation)	3516						
No	2843	2783 (80·8)	60 (83·3)	ref		ref	
Yes	673	661 (19·2)	12 (16·7)	0·87 (0·47, 1·61)	0·654	1.00 (0·55, 1·83)	0·994
Loss of pregnancy (<20 weeks)[2]	860						
No	850	831 (98·8)	19 (100)	ref		ref	
Yes	10	10 (1·2)	0 (10)	-	-	-	-
Stillbirth (≥20 weeks gestation)	860						
No	833	815 (96·9)	18 (94·7)	ref		ref	
Yes	27	26 (3·1)	1 (5·3)	1·70 (0·19, 14·99)	0·632	1·45 (0·17, 12·21)	0·730
Birthweight (kilograms)	2235	3·4 (3·0, 3·6)	3·2 (3·0, 3·4)	-0·16 (-0·24, -0·08)	0·001	-0·18 (-0·28, -0·09)	0·001
Birthweight (<2·5 kilograms)[2]	2235						
No	2185	2110 (97·7)	75 (100)	ref		ref	
Yes	50	50 (2·3)	0 (0)	-	-	-	-
Birth length (centimeter)	853	50·0 (50·0, 52·0)	50·0 (50·0, 54·0)	0·50 (-1·50, 2·24)	0.558	0·69 (-0·93, 2·30)	0.383
Small for gestational age	2231						
No	1989	1944 (90·2)	45 (84·9)	ref		ref	
Yes	220	212 (9·8)	8 (15·1)	1·54 (0·72, 3·29)	0·270	1·56 (0·73, 3·32)	0·248
Congenital malformation[2]	3499						
No	3483	3414 (99·5)	69 (100·0)	ref		ref	
Yes	16	16 (0·5)	0 (0·0)	-	-	-	-
Neonatal death	3530						
No	3474	3375 (98·4)	68 (98·6)	ref		ref	
Yes	56	55 (1·6)	1 (1·0)	0·904 (0·12, 6·76)	0·922	1·00 (0·13, 7·71)	0·999

[1]Tenofovir-Diphosphate (TFV-DP) concentrations ≥ 200 fmol/punch, consistent with ≥ 2 doses oral TDF/FTC based PrEP according to adherence benchmarks established in IMPAACT 2009 for pregnant women as measured in dried blood spots
[2]Adjusted for maternal age, primigravida, gestational age, partner HIV status, and syphilis status at enrollment
[3]Prevalence ratios shown for binary outcomes and beta-coefficients shown for continuous outcomes. PR and P-value not shown due to 0% prevalence among PrEP-exposed

Supplemental Table 6· Infant growth outcomes at 6-weeks, 6-months, and 9-months postpartum by confirmed PrEP exposure ≥ 2 doses/wk

		Median (IQR) or n (%)		Unadjusted GEE regression		Adjusted GEE regression[2]	
		Quantified PrEP exposure ≥2 doses/week during pregnancy[1]					
	N (n=3516)	No (n=3505)	Yes (n=72)	Coeff[3] (95% CI)	*p*	Coeff[3] (95% CI)	*p*
**6-Weeks**							
Weight (kg)	2225	5·0 (4·5, 5·4)	5·1 (4·5, 5·7)	0·11 (-0·07, 0·30)	0·222	0·08 (-0·13, 0·29)	0·447
Absolute WAZ	2194	0·3 (-0·4, 0·9)	0·6 (-0·4, 1·1)	0·22 (-0·02, 0·46)	0·072	0·17 (-0·11, 0·45)	0·215
Underweight (<-2 WAZ)[2]	2194						
No	2129	2086 (97·1)	43 (100·0)	ref		ref	
Yes	65	65 (2·9)	0 (0·0)	-	-	-	-
Length (cm)	2193	55·0 (54·0, 57·0)	56·0 (54·0, 57·6)	-0·46 (-2·73, 1·80)	0·673	-0·54 (-2·84, 1·75)	0·627
Absolute LAZ	2166	-0·2 (-1·1, 0·7)	0·0 (-0·4, 0·9)	0·33 (-0·06, 0·72)	0·090	0·28 (-0·10, 0·66)	0·145
Stunting (<-2 LAZ)	2166						
No	1945	1907 (90·5)	38 (92·7)	ref		ref	
Yes	204	201 (9·5)	3 (7·3)	0·77 (0·32, 1·84)	0·553	0.99(0·39, 2·49)	0·978
Absolute WLZ	2111	0·6 (-0·3, 1·7)	0·6 (-0·3, 1·6)	-0·02 (-0·40, 0·35)	0·893	-0·00 (-0·38, 0·38)	1·000
Wasting (<-2 WLZ)	2111						
No	1988	1949 (94·2)	39 (93·0)	ref		ref	
Yes	123	121 (5·8)	2 (4·9)	0·83 (0·21, 3·31)	0·797	0·78 (0·20, 2·98)	0·711
**6-Months**							
Weight (kg)	1872	7·7 (7·0, 8·5)	7·6 (7·0, 8·2)	-0·04 (-0·42, 0·34)	0·828	-0·043 (-0·46, 0·37)	0·833
Absolute WAZ	1782	0·1 (-0·7, 0·9)	-0·2 (-0·7, 0·8)	-0·04 (-0·42, 0·34)	0·819	-0·03 (-0·45, 0·39)	0·886
Underweight (<-2 WAZ)	1782						
No	1722	1678 (96·6)	44 (98·2)	ref		ref	
Yes	60	59 (3·4)	1 (1·8)	0·65 (0·11, 3·81)	0·637	0·59 (0·09, 3·77)	0·579
Length (cm)	1877	66·0 (64·0, 68·0)	66·0 (64·0, 69·0)	0·3151 (-0·74, 1·04)	0·728	0·04 (-1·00, 1·07)	0·941
Absolute LAZ	1769	-0·3 (-1·1, 0·6)	-0·3 (-1·2, 0·6)	-0·27 (-0·67, 0·13)	0·168	-0·32 (-0·73, 0·10)	0·128
Stunting (<-2 LAZ)	1769						
No	1613	1575 (91·3)	38 (87·5)	ref		ref	
Yes	156	150 (8·7)	6 (12·5)	1·57 (0·68, 3·62)	0·362	1·77 (0·83, 3·81)	0·141
Absolute WLZ	1766	0·4 (-0·5, 1·4)	0·1 (-0·5, 1·5)	0·05 (-0·28, 0·37)	0·775	0·09 (-0·26, 0·44)	0·580

Wasting (<-2 WLZ)	1766						
No	1688	1645 (96·1)	43 (100·0)	ref		ref	
Yes	67	67 (3·9)	0 (0·0)	-	-	-	-
**9-Months**							
Weight (kg)	1887	8·6 (7·9, 9·6)	8·5 (7·9, 9·2)	-0·02 (-0·39, 0·35)	0·908	0·00 (-0·36, 0·36)	0·998
Absolute WAZ	1784	0·1 (-0·7, 1·0)	-0·2 (-0·8, 0·8)	-0·04 (-0·36, 0·28)	0·793	-0·05 (-0·34, 0·23)	0·693
Underweight (<-2 WAZ)	1784						
No	1716	1671 (96·1)	45 (97·9)	ref		ref	
Yes	68	67 (3·9)	1 (2·2)	0·56 (0·08, 3·82)	0·557	0·51 (0·08, 3·23)	0·474
Length (cm)	1829	70·0 (68·0, 72·0)	71·0 (68·8, 72·0)	0·78 (-0·12, 1·68)	0·086	0·47 (-0·66, 1·60)	0·396
Absolute LAZ	1747	-0·4 (-1·2, 0·5)	-0·3 (-1·1, 0·7)	0·10 (-0·24, 0·43)	0·554	0·0 (-0·31, 0·4322)	0·974
Stunting (<-2 LAZ)	1747						
No	1588	1544 (90·8)	44 (95·7)	ref		ref	
Yes	159	157 (9·2)	2 (4·3)	0·47 (0·07, 3·21)	0·442	0·56 (0·08, 3·90)	0·557
Absolute WLZ	1737	0·4 (-0·5, 1·3)	0·2 (-0·6, 1·1)	-0·11 (-0·46, 0·23)	0·498	-0·04 (-0·38, 0·30)	0·811
Wasting (<-2 WLZ)	1737						
No	1675	1631 (96·5)	44 (95·7)	ref		ref	
Yes	62	60 (3·5)	2 (4·3)	1·23 (0·33, 4·55)	0·762	0·97 (0·27, 3·48)	0·969

PrEP=pre-exposure prophylaxis. P=p-value. LAZ=length-for-age Z score. WAZ=weight-for-age Z score. WLZ=weight-for-length Z score.

[1]Tenofovir-Diphosphate (TFV-DP) concentrations ≥ 200 fmol/punch, consistent with ≥ 2 doses oral TDF/FTC based PrEP according to adherence benchmarks established in IMPAACT 2009 for pregnant women as measured in dried blood spots

[2]Adjusted for maternal age, primigravida, gestational age, partner HIV status, and syphilis status at enrollment

[3]Prevalence ratios shown for binary outcomes and beta-coefficients shown for continuous outcomes. PR and P-value not shown due to 0% prevalence among PrEP-exposed

Appendix. PrIMA Study Team

Alphonce Onyango Kungu

Asterico Neema

Ben Ochieng Odhiambo

Benard Andiego Ouma

Bilha Anyango Aomo

Conceptor Anyango

Daisy Kemunto Mong'are

Dan Otieno Ngicho

Daniel Odinga

David Abiero

Deckins Agwanda

Diana Akinyi Oreje

Donvix Olwok

Elizabeth Adongo

Emma Akello Opoko

Emmaculate Mukenyi

Enock Sifuna Wawire

Eunice Anyiego

Everlyne Otumba

Fredrick OwinoAndere

Frida Ondiek

Geoffrey Gichana Maiko

George Oketch

George Ouma

Gift Akinyi Atieno

Grace Furnace

Gradus Oduor

Herbert Ogayo

Irene Ouma

James Ingutia

Jane Atieno Okoth

Joan Akoth Ng'ere

Joshua Onyango

Judith Nyakina

Kenneth Odede Ojwang

Leakey Odongo

Linda Merab Orwa

Lucas Nyahuru

Lukio Agalo Obel

Maureen Anyango

Maureen Oketch

Maurice Mbogori

Maurine Akoth Okoth

Michael Abuong

Moses Fidah Kirani

Nancy Oboke

Nereah Awuor Ojiem

Pascal Otieno

Pauline Achieng Juma

Peter Kennedy Oloo

Rhoda Oricho

Richard Omondi

Rita Akumu

Rita Nawire

Robert Ochieng Oliech

Sara Awino Ayuyo

Sarah Achieng Oketch

Scholastica Adhiambo

Sharon Achieng Oloo

Sheilah Akinyi Marienga

Stephenie L. Ojiambo

Verena Okunya

Violet Achieng Apondi

Vivian Chepkemoi

Wallace Juma

Wilkista Awiti

Winnie Mituga

Affiliation: Kenyatta National Hospital
