# Supplementary material for: Prenatal exposure to HIV pre-exposure prophylaxis and birth, growth, and social–emotional developmental outcomes throughout early childhood in Kenya: a prospective cohort study
Source: Lancet Glob Health. Author manuscript; Available in PMC 2025 Apr 3. (PMC11964894; doi:10.1016/S2214-109X(24)00471-6)
Supplement: 3 [file NIHMS2061315-supplement-3.pdf]

THE LANCET
Global Health

Supplementary appendix 3

This Equitable Partnership Declaration (EPD) was submitted by the authors, and we reproduce it as supplied. It has not been peer reviewed. *The Lancet*'s editorial processes have not been applied to the EPD.

Supplement to: Gómez L, Kinuthia J, Abuna F, et al. Prenatal exposure to HIV pre-exposure prophylaxis and birth, growth, and social–emotional developmental outcomes throughout early childhood in Kenya: a prospective cohort study. *Lancet Glob Health* 2025; **13:** e467–78.

<u>Equitable Partnership Declaration questions</u>

<u>Researcher considerations</u>

1. Please detail the involvement that researchers who are based in the region(s) of study had during a) study design; b) clinical study processes, such as processing blood samples, prescribing medication, or patient recruitment; c) data interpretation; and d) manuscript preparation, commenting on all aspects. If they were not involved in any of these aspects, please explain why.

   This question is intended for international partnerships; if all your authors are based in the area of study, this question is not applicable.

   This should include a thorough description of their leadership role(s) in the study. Are local researchers named in the author list or the acknowledgements, or are they not mentioned at all (and, if not, why)? Please also describe the involvement of early career researchers based in the location of the study. Some of this information might be repeated from the Contributors section in the manuscript. Note: we adhere to ICMJE authorship criteria when deciding who should be named on a paper.

**a) Study design:**<br>The Kenyan site principal investigator (PI), had input in the conception and design of the study, contributing valuable insights and expertise specific to the local context. He is included as the second author of this manuscript. The Kenyan site PI was first author on the primary manuscript from the parent study (Kinuthia et al. *JIAS* 2023).
**b) Clinical study processes:**<br>The Kenyan site PI had clinical oversight of all study sites, study staff, and study procedures The Kenyan Project Director, who reports directly to the site PI, was instrumental in the implementation and adherence to clinical study procedures. In-country study staff (nurses and retention officers) conducted all participant recruitment, enrolment, and follow-up procedures, including dispensing PrEP, collecting lab specimens, and conducting antenatal and postnatal care visits. These study staff are referred to in the author list as the PrIMA study team and are mentioned in the acknowledgements. The Kenyan lab manager oversaw transportation and storage of all laboratory specimens and is named in the author list. Kenyan research coordinators facilitated and supported clinical study processes and are named on the author list.
**c) Data interpretation:**<br>Kenyan data managers were involved in routine data monitoring, cleaning and report generation and data interpretation and are named in the author list.
**d) Manuscript preparation:**<br>All of the above personnel were involved in the manuscript preparation, including providing input and suggestions on presentation of materials.

2. Were the data used in your study collected by authors named on the paper, or have they been extracted from a source such as a national survey? ie, is this a secondary analysis of data that were not collected by the authors of this paper. If the authors of this paper were not involved in data collection, how were data interpreted with sufficient contextual knowledge?

The Lancet Global Health *believe contextual understanding is crucial for informed data analysis and interpretation.*

Data for this study were collected directly by the authors named in the manuscript. The direct involvement of local researchers in the design and data collection process ensured a deep contextual understanding of the local setting, allowing for more nuanced and informed analysis and interpretation of the findings.

3. How was funding used to remunerate and enhance the skills of researchers and institutions based in the area(s) of study? And how was funding used to improve research infrastructure in the area of study?

   Potentially effective investments into long-term skills and opportunities within institutions could include training or mentorship in analytical techniques and manuscript writing, opportunities to lead all or specific aspects of the study, financial remuneration rather than requiring volunteers, and other professional development and educational opportunities.

   Improvements to research infrastructure could be funding of extended trial designs (such as platform trials) and use of master protocols to enable these designs, establishment of long-term contracts for research staff, building research facilities, and local control of funding allocation.

Skills:

Training opportunities for staff: Throughout the study, we actively worked to identify and support training opportunities for all Kenyan team members. These experiences served to build capacity for team members and also provided professional development opportunities.

- The study sponsored 21 Kenyan study staff members (6 nurses, 6 retention officers, 2 data managers, 2 lab managers, 3 study administrators, 1 research coordinator, 1 assistant study coordinator) to participate in several 10-week online professional development courses offered by the University of Washington's Global Health E-Learning Program (eDGH). The online courses consisted of video lectures, readings, discussion forums, quizzes, case studies and assignments. Courses offered included: Introduction to Epidemiology for Global Health, Global Mental Health, and Project Management in Global Health.
- The project sponsored two staff from Kenya to attend the University of Washington's Principles of STI/HIV Research and Public Health Practice Course, an annual intensive two-week cross-disciplinary training. The course provides an overview of behavioral, clinical, epidemiological, statistical, operational, and pathogenesis research in STD/HIV.

- The project supported two Kenyan staff trainees who matriculated as graduate students into the University of Washington's School of Public Health's Master of Public Health program into the Fogarty-funded, year-long training program in which mentees based in Kenya develop research projects within the current study and receive mentorship from study co-investigators. Students received training and mentoring on ethical conduct of research, study design, and analytical methods and developed a thesis using study data.
- Dr. Pintye led several intensive manuscript writing workshops that included 6 Kenyan staff members and involved practical based training on developing a research question, literature review, shell tables, and all the sections of a manuscript. Those with no or limited manuscript writing experience were matched with a mentor as they progressed with manuscript development. Following these workshops, several Kenyan staff presented abstracts at international conference and submitted manuscripts to academic journals.
- The study conducted several rounds of continuing medical education (CME) activities at all study facilities to engage the facility staff in learning more about PrEP. Outside of formal CME settings, PrIMA staff have also provided technical assistance to facility staff on PrEP implementation.

Research infrastructure:

- The study supported PrEP delivery at all study sites and created infrastructure to allow for delivery of PrEP within Maternal Child Health Clinics. Prior to the study PrEP was only available in the Comprehensive Care Clinics. Upon study completion, study staff re-trained existing clinic staff on the delivery of PrEP to ensure sustainability of PrEP delivery in MCH.
- The study supported the expansion of MCH clinics in several study sites, including the procurement of tenting structures.
- The study procured equipment to improve laboratory operations including freezers, refrigerators, and cabinets.

4. How did you safeguard the researchers who implemented the study?

Please describe how you guaranteed safe working conditions for study staff, including provision of appropriate personal protective equipment, protection from violence, and prevention of overworking.

During the COVID-19 pandemic the study followed guidelines from the University of Washington Human Subjects Division (UW HSD), the Kenyatta National Hospital Ethics and Research Committee (KNH ERC) and the Kenya Ministry of Health (MoH) to ensure the safety of all participants, staff, and the community. During the pandemic additional provisions to reduce risk included:

- Introduction of handwashing and symptom screening/triage stations each facility's main gates in accordance with MoH recommendations
- Procurement of PPE for study staff and MCH clinics within study facilities
- Donning appropriate PPE for client interaction as recommended by MoH, maintaining physical distancing as possible during visits and performing proper sanitation and hygiene between interactions
- Suspension of home visits
- Rescheduling study visits that do not align with clinical care visits or for clients experiencing respiratory symptoms
- Maintaining staff symptom monitoring and return to work
- Developed contingency plans for at risk-staff including job expectations and potential alternate roles and locations.

- Held special trainings for COVID-19, including:
    - Proper disinfection protocols (from entryway to exit, define janitorial, clinician, and lab staff duties)
    - Infection control precautions
    - Proper use of PPE for patients and staff (including fit checking, applying simple mask to patients with cough before escorting them out, and hand hygiene).

Benefits to the communities and regions of study

5. How does the study address the research and policy priorities of its location?

How were the local priorities determined and then used to inform the research question? Who decided which priorities to take forward? Which elements of the study address those priorities?

Kenya was an early adopter of PrEP regionally and the Ministry of Health approved use of PrEP among individuals who are pregnant and breastfeeding in 2016. Our team worked closely with Kenya Ministry of Health as part of study development and initiation activities. Dr. John Kinuthia, the study's Kenya-based site PI, is a member of national technical working groups related to the elimination of mother-to-child transmission of HIV (eMTCT) efforts. This has resulted in active engagement of county and national-level MoH officials to ensure that the study protocol and approaches align with the government's research priorities. Mr. Felix Otieno (Project Director) and Dr. Kinuthia regularly conduct meetings with stakeholders at the national and county level to sensitize them to the study and disseminate study findings. These meetings have been critical in preparing for study initiation, including facilitating training material development and development of dissemination materials.

6. How will research products be shared in the community of study?

For instance, will you be providing written or oral layperson summaries for non-academic information sharing? Will study data be made available to institutions in the region(s) of study? The Lancet Global Health *encourages authors to translate the summary (abstract) into relevant languages after paper editing; do you intend to translate your summary?*

National Kenyan Stakeholders

We will continue to present findings to Kenyan stakeholders through a variety of channels, including:

- Presentations at Kenyan conferences such as the University of Nairobi Collaborative HIV Research Conference
- Presentations at the Kenya National NASCOP PrEP technical working groups.
- One-on-one meetings with key stakeholders including:
    - Program Managers for Prevention of Mother-to-Child Transmission and PrEP programs within the Kenyan Ministry of Health
    - Other PrEP implementing partners from around Kenya

Kenyan County Stakeholders

Dissemination within Siaya and Homa Bay counties is ongoing, and will continue to expand as the project comes to an end. Specific activities include:

- Annual community advisory board meetings in both Siaya and Homa Bay counties
- Annual meetings with the health management teams of Siaya and Homa Bay counties to sensitize them to the PrIMA study and results as they are available.
- Continuing medical education trainings at each of the study facilities to sensitize facility staff to the study, increase knowledge and awareness of PrEP, and present study results.

We would like to translate our abstract to Swahili.

7. How were individuals, communities, and environments protected from harm?

a) *How did you ensure that sensitive patient data was handled safely and respectfully? Was there any potential for stigma or discrimination against participants arising from any of the procedures or outcomes of the study?*

Confidentiality is of critical importance, and we took many precautions to protect against the possibility of a breach of confidentiality. Disclosure of medical or other personal information, particularly related to HIV prevention, may pose personal or social risks, especially within community and family settings. Our research team is very aware of the importance of maintaining strict confidentiality and has extensive experience dealing with sensitive health information from working in this field for the past 25 years. The following precautions were set to protect the privacy of participants and maintain confidentiality of research data:

1. All study staff will be well trained and will receive ongoing supervision in confidentiality and data security procedures, specifically in ethical conduct, confidentiality protection, mandated reporting, and other topics of human participant protection. In addition, the importance of confidentiality will be emphasized with clinical staff who may have knowledge about patients' eligibility or participation in the study.
2. As part of the consent procedure, participants will be informed of the limits of confidentiality (harm to self or others) and mandated reporting requirements.
3. Privacy will be maintained by conducting all interviews, discussions, study assessments, and study procedures in closed and private rooms.
4. All study-related information will be stored securely at the study site. The study office is considered a secure data storage location. Forms, lists, logbooks, appointment books, and any other listings that link participant ID numbers to other identifying information will be stored in a separate, locked file in an area with limited access.
5. Data (including locator information, etc) will be securely stored in separate locked file cabinets and in password protected documents on password-protected computers and secure servers. Access to data storage areas and computers will be restricted.
6. Only deidentified data will be sent to investigators at University of Washington, where it will be stored on a secure server, accessible only through password-protected computers with encryption.
7. Analysis will occur only on deidentified data.
8. Data will only be stored for as long as necessary to complete the study, and for adherence to IRB regulations.
9. The information gathered will be used only for scientific, educational, or instructional purposes.
10. Data collection, administrative forms, laboratory specimens, and other reports will be identified only by a coded number to maintain participant confidentiality. Thus, while we acknowledge that a breach of confidentiality is possible, the likelihood is very low.

b) *Might any of the tests be experienced as invasive or culturally insensitive?*

No

c) *How did you determine that work was sensitive to traditions, restrictions, and considerations of all cultural and religious groups in the study population?*

We convened a community advisory board (CAB) that includes local representation from Siaya and Homa Bay counties to review and advise on study procedures and address any community concerns. CAB members include community health volunteers, religious leaders, teachers, youth, women, MCH clinicians, and county representatives.

d) *Were biowaste and radioactive waste disposed of in accordance with local laws?*

Yes

e) *Were any structures built that would have impacted members of the community or the environment (such as handwashing facilities in a public space)? If so, how did you ensure that you had appropriate community buy-in?*

Enhancements to facilities were supported by the study at the request of the local facility administration and local officials.

f) *How might the study have impacted existing health-care resources (such as staff workloads, use of equipment that is typically employed elsewhere, or reallocation of public funds)?*

The study supported PrEP delivery at all study sites and created infrastructure to allow for delivery of PrEP within Maternal Child Health Clinics. Prior to the study PrEP was only available in the Comprehensive Care Clinics. Upon study completion, study staff re-trained existing clinic staff on the delivery of PrEP to ensure sustainability of PrEP delivery in MCH.
The study reduced facility staff workloads by integrating study staffs within the facility and providing their services within the MCH, engaging patients and study participants.

8. Finally, please provide the title (eg, Dr/Prof, Mr/Mrs/Ms/Mx), name, and email address of an author who can be contacted about this statement. This can be the corresponding author.

Name: Dr. Jillian Pintye
Email: jpintye@uw.edu
